# Cardiovascular risk factors and kidney function among automobile mechanic and their association with serum heavy metals in Southwest Nigeria: A cross-sectional study

**DOI:** 10.1371/journal.pone.0292364

**Published:** 2023-10-11

**Authors:** Oluseyi Ademola Adejumo, Adenike Christianah Enikuomehin, Adeyemi Ogunleye, Walter Bamikole Osungbemiro, Alex Adedotun Adelosoye, Ayodeji Akinwumi Akinbodewa, Olutoyin Morenike Lawal, Stanley Chidozie Ngoka, Oladimeji Adedeji Junaid, Kenechukwu Okonkwo, Emmanuel Oladimeji Alli, Rasheed Olanshile Oloyede

**Affiliations:** 1 Department of Internal Medicine, University of Medical Sciences, Ondo City, Ondo State, Nigeria; 2 Department of Medical Laboratory Science, University of Medical Sciences, Ondo City, Ondo State, Nigeria; 3 Department of Chemistry, University of Medical Sciences, Ondo City, Ondo State, Nigeria; 4 Department of Family Medicine, University of Medical Sciences Teaching Hospital, Akure, Ondo State, Nigeria; 5 Department of Internal Medicine, Federal University Teaching Hospital, Owerri, Imo State, Nigeria; Obafemi Awolowo University, NIGERIA

## Abstract

**Introduction:**

The burden of cardiovascular disease (CVD) is huge due to its associated morbidity, mortality and adverse socio-economic impact. Environmental pollution as a risk factor contributes significantly to the burden of CVD, especially in the low and middle income countries. One of the effective strategies to reduce CVD burden is to prevent or detect cardiovascular risk factors early in at-risk population. This study determined some cardiovascular risk factors, kidney function, and their association with heavy metals among automobile mechanics.

**Method:**

This was a cross-sectional study involving 162 automobile mechanics and 81 age and sex matched controls. Serum levels of lead, cadmium and some cardiovascular risks were assessed and compared in the two groups. Associations between serum lead, cadmium and some cardiovascular risks were determined using correlation analysis. P value of <0.05 was taken as significant.

**Results:**

The mean ages of the automobile mechanics and controls were 47.27±9.99 years and 48.94±10.34 years, respectively. The prevalence of elevated serum cadmium was significantly higher in the automobile mechanics (25.9% vs 7.9%; p = <0.001). The significant cardiovascular risk factors in the automobile mechanics vs controls were elevated total cholesterol (32.1% vs 18.5%; p = 0.017), hyperuricemia (20.4% vs 1.2%; p = <0.001), elevated blood glucose (16.0% vs 4.9% p = 0.013); and alcohol use (55.1% vs 30.0%; p = 0.001). Among the automobile mechanics, there were significant positive correlations between serum cadmium, atherogenic index of plasma (AIP) (p = 0.024; r = 0.382) and triglyceride (p = 0.020; r = 0.391). Significant positive correlation was found between serum lead and neutrophil gelatinase associated lipocalin (NGAL) (p = <0.001; r = 0.329). There were significant positive correlation between serum cadmium level, AIP (p = 0.016; r = 0.373) and TG (p = 0.004; r = 0.439); between serum lead and NGAL in all the study participants (p = 0.005; r = 0.206).

**Conclusion:**

Automobile mechanics have notable exposure to heavy metals and a higher prevalence of some cardiovascular risk factors. Health education and sensitisation as well as policies that would regulate exposure of persons to heavy metals should be implemented in Nigeria.

## Introduction

Cardiovascular disease (CVD) is a disease of public health importance due to its increasing prevalence, associated morbidity, mortality and socio-economic impact [1–3]. CVD accounts for 17.9 million deaths globally. The burden of CVD is however, higher in low and middle-income countries (LMIC) [1, 2, 4]. Cardiovascular risk factors which account for CVD can be either traditional or novel. These risk factors include hypertension, diabetes mellitus, tobacco use, dyslipidaemia, obesity, depression, environmental pollution, low education, low grip strength and poor diet [1, 4]. The contribution of cardiovascular risk factors as drivers of cardiovascular morbidities and mortality varies in different countries [1, 4]. Prevention or early detection of cardiovascular risk factors constitute one of the major strategies to reduce the burden of CVD. Of the risk factors stated above, environmental pollution and contamination is a more common contributor to CVD in LMIC compared to high income countries [1, 4]. Occupational practices of artisans such as automobile mechanics may contribute to environmental pollution and contamination [5–7]. Previous studies within and outside Nigeria have reported higher serum levels of some heavy metals in automobile mechanics [8–10]. Hazardous occupational practices such as regular use of diesel and petrol to wash the hands and feet as well as constant oral sucking of fuel may partly account for higher levels of some heavy metals in automobile mechanics [11]. The report of Akpoveta et al [12] specifically showed higher concentration of heavy metals such as lead in diesel and petrol in Nigeria beyond the generally permissible levels in refined petroleum products.

Although, the pathophysiology of association between heavy metal exposure and CVD is not well understood, both experimental and human studies have implicated the activation of renin-angiotensin-aldosterone system, generation of reactive oxygen species and inflammation [13]. Heavy metals have also been found to be associated with increased prevalence of some cardiovascular risk factors such hypertension, hyperuricaemia and dyslipidaemia [13].

There is limited information on the relationship between heavy metals, cardiovascular risk and kidney function among those that are occupationally exposed in Nigeria. Most screening programs that aimed at reducing cardiovascular disease and kidney disease burden tend to pay less attention of those whose occupation increases their risk of developing these diseases. This study aimed to fill part of this existing gap by determining some cardiovascular risk factors, kidney function, and their association with heavy metals among automobile mechanics. The findings of this study will also provide scientific evidence to advocate for safe occupational practices among automobile mechanics.

## Methodology

This was a cross-sectional study that was carried out in Ondo State, Nigeria between November 2021 and February, 2022. Ondo state is one of the 36 states in Nigeria and is located in the Southwestern part of the country.

### Study population

The study participants comprised of automobile mechanics in Ondo and Akure city, Ondo State. Inclusion criteria were automobile mechanic with a minimum working experience of 1 year, aged ≥ 18 years, and those who gave informed consent. Any automobile mechanic with known kidney disease, heart disease or liver disease based on history and physical examination was excluded from participating in the study. Controls were individuals who were not automobile mechanics and have not had significant exposure to petrochemicals.

### Sample size determination

The sample size was determined using the formula for single proportion [14]. The prevalence of a cardiovascular risk factor (generalized obesity) among automobile mechanic used in this calculation was 5.8% based on report from a previous study [15]. The confidence interval was taken as 95% and the power of the study was 80%. The minimum sample size for this study was 92 after including 10% attrition. A total of 182 automobile mechanics and 91 age and sex matched controls who were not automobile mechanics were consecutively recruited in the study.

### Study procedure

Questionnaire was administered by the researchers to study participants to obtain socio-demographic information; occupational history such as number of years of practice; use of protective overall gown during work; occupational practices relating to the use of petrochemicals; exposure to petrol and diesel; medical history; and social history such as smoking and alcohol use. Interview was conducted in Yoruba language for the respondents who did not understand English language. All the study participants completed the questionnaires and underwent physical examination.

Height was measured in metres (m) to the nearest 0.1m using a graduated height scale with the participant in erect position without shoes. Weight was measured in kilogram (kg) to the nearest 0.1kg using standard weighing scale with participants wearing light clothing without shoes, cap or headgear. Body mass index (BMI) was calculated as follow;

BMI (kg/m^2^) = weight (kg)/height^2^(m^2^).

The waist circumference was measured using inelastic tape to the nearest 0.1cm, in the horizontal plane, mid-way between the anterior margin of the lowest rib and the iliac crest with the participant standing comfortably and at the end of normal expiration. Blood pressure was measured using Accoson mercury sphygmomanometer. This was taken with participant comfortably seated with arm rested on a table.

Ten ml of fasting blood samples was collected from the study participants for serum lead, cadmium, fasting serum lipid profile, uric acid, total antioxidant capacity (TAC), neutrophil gelatinase-associated lipocalin (NGAL), creatinine and blood glucose. Five ml of spot urine was collected and analyzed for albumin-creatinine ratio (ACR). ACR was determined using appropriate spectrophotometric methods [16]. Heavy metals levels was determined using atomic absorption spectroscopy.

#### Blood sample preparation and digestion for heavy metal analysis

A modification of the procedure used by Uddin et al [17] was adopted for the digestion of the blood sample. 3 ml of whole blood sample was placed in a test tube, and a 5 ml mixture of nitric-hydrochloric acid (HNO_3_–HCl) was added to the test tube in a ratio 3:1. The test tube was heated on a hot plate at 100°C for 3 hours until a clear sample was obtained. After cooling, the sample was then filtered using a 0.45 μm filter paper. The filtrate was made up to 10 mL with deionized water and stored for elemental analysis using an Atomic Absorption Spectrophotometer (Buck 200 model).

TAC was estimated using standard spectrophotometric method based on the ferric reducing antioxidant power (FRAP) assay [18]. 1.5 mL of freshly constituted solutions of 2,4,6-tripyridyl-s-triazine and ferric chloride hexahydrate were made to react with 50μL of sample at 37°C and the result expressed in μmol Trolox equiv/L. Malondialdehyde (MDA) was measured spectrophotometrically based on its reaction with 2-thiobarturic acid (TBA) in acidic pH. This reaction was measured and MDA value calculated [19]. Plasma NGAL was analyzed using Sandwich-ELISA kit produced by the Elabscience US.

#### Definition of terms

Central obesity was defined as waist circumference ≥ 102cm for males [20]. Generalized obesity was defined as BMI ≥30 kg/m^2^ [21]. Elevated total cholesterol was defined as TC > 5.17mmol/l; low high-density lipoprotein cholesterol (HDL-C) was defined as HDL-C < 1.03mmol/l; elevated low-density lipoprotein cholesterol (LDL-C) was defined as LDL-C >3.40mmol/l; and elevated triglyceride (TG) was defined as TG >1.70mmol/l [22]. Normoalbuminuria was defined as ACR value <30mg/g, microalbuminuria was defined as ACR value between 30-299mg/g and macroalbuminuria was defined as ACR value ≥300mg/g [23]. Reduced glomerular filtration rate (GFR) was defined as a GFR of less than 60mls/mins/1.73m^2^ [23]. Elevated serum cadmium was taken as cadmium level above the permissible concentration of 0.03–0.12 μg/dl or 0.0003–0.0012 mg/L [24]. Elevated serum lead was taken as lead level greater than the allowed concentration of 0–10 *μ*g/dL or 0–0.1 mg/L [25]. The atherogenic index of plasma (AIP) was defined as the logarithm of the ratio TG and HDL-C (log TG/HDL-C) [26]. AIP value >0.24 was defined as high cardiovascular risk [26].

#### Ethical consideration

Informed consent was obtained from all participants and the information was treated with utmost confidentiality. Ethical approval with reference number UNIMED/HREC/OndoSt/22/06/21 was obtained from the Human Research and Ethics Committee of the University of Medical Sciences, Ondo State.

#### Statistical analysis

Data obtained were entered and analyzed using the Statistical Package for Social Sciences (SPSS) version 21.0 software 9 (IBM-SPSS, Armonk, NY: IBM Corporation). Descriptive data were presented as tables and categorical variables of the two groups (automobile mechanics and controls) were expressed as proportions and percentages. Normally distributed data were presented as mean and standard deviation while skewed data were presented as median and interquartile range. Association between categorical variables was analyzed using Chi-square. Fisher’s exact test was used when the number of counts was less than 5. Correlation was used to determine association between continuous variables. The level of significance for each test was set at p < 0.05.

## Results

There were 243 male participants in this study comprising of 162 auto-mechanics and 81 age and sex matched controls. The mean age of the automobile mechanics and the controls were 47.27±9.99 years and 48.94±10.34 years, respectively. One-hundred and thirteen (69.8%) of the automobilemechanics were between the age group of 40–60 years and Christians. Majority (92%) of the automobile mechanics were married. The controls were significantly more educated (p = <0.001) than the automobilemechanics. Ninety-seven (60%) of the automobile mechanics had below 10 years working experience Table 1.

**Table 1:** Socio-demographic characteristics of study participants.

Variables	Automobile Mechanic Group n = 162	Control Group n = 81	p-value
	Frequency (%)	Frequency (%)	
Age			
Mean Age	47.27±9.99	48.94±10.34	
<40 years	27(16.7)	13(16.0)	0.992
40–60 years	113(69.8)	57(70.4)	
≥61 years	22(13.5)	11(13.6)	
Gender			
Male	162(100)	81(100)	
Marital Status			
Single	10(6.2)	6(7.4)	0.915
Married	149(92.0)	74(91.4)	
Divorced	3(1.8)	1(1.2)	
Religion			
Christianity	113(69.8)	80(98.6)	<0.001
Islam	48(29.6)	1(1.4)	
Others	1(0.6)	0(0)	
Educational Level			
None	3(1.9)	1(1.4)	<0.001
Primary	55(33.9)	6(7.1)	
Secondary	92(56.8)	21(25.7)	
Tertiary	12(7.4)	53(65.7)	
Working Experience			
<10 years	97(59.9)		
10–20 years	38(23.5)		
>20 years	27(16.7)		

**Fig 1:**
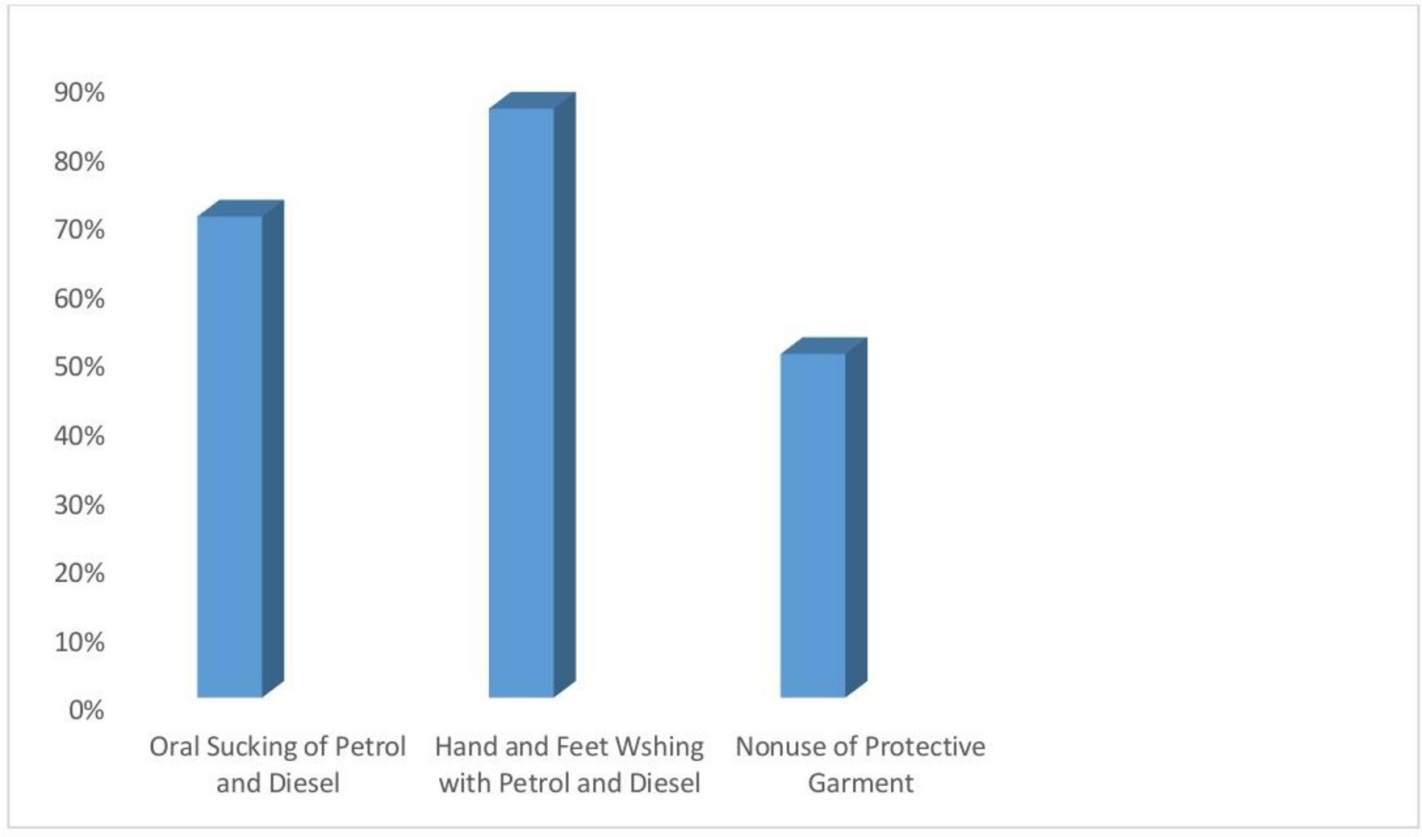
Harmful occupational practices among automobile mechanics.

While at work, about 50% of the auto-mechanics regularly wore overall coat, 139 (85.8%) frequently used petrol and diesel to wash their hands and feet and 117 (72.2%) regularly sucked petrol with mouth Fig 1.

The prevalence of elevated TC (32.1% vs 18.5%; p = 0.017), hyperuricemia (20.4% vs 1.2%; p = <0.001), elevated blood glucose (16.0% vs 4.9% p = 0.013); and alcohol use (55.1% vs 30.0%; p = 0.001) were significantly higher in the automobilemechanics compared to the control group. A significantly higher proportion of automobile mechanics had high serum cadmium level compared to the controls (25.3% vs 7.4%; p = <0.001). There was no significant difference in the proportion of automobile mechanics and controls with high serum lead level (82.7% vs 77.8%; p = 0.354) Table 2.

**Table 2:** Comparison of cardiovascular risk between the automobile mechanics and controls.

	Auto-mechanic Group n = 162	Control Group n = 81	P-value
	Frequency (%)	Frequency (%)	
Elevated Serum Cadmium	41(25.3)	6(7.4)	0.002
Elevated Serum Lead	134(82.7)	63(77.8)	0.363
High LDL-C	52(32.1)	19(23.5)	0.105
Low HDL-C	57(35.2)	31(38.3)	0.369
High TC	52(32.1)	15(18.5)	0.017
High TG	30(18.5)	8(9.9)	0.093
Hyperuricemia	33(20.4)	1(1.2)	<0.001
Elevated Blood Glucose	26(16.1)	4(4.9)	0.013
Hypertension	57(35.2)	39(48.2)	0.070
Obesity/Overweight	66(40.7)	33(40.7)	1.00
Abdominal obesity	16(9.9)	8(9.9)	1.00
Reduce GFR	43(26.5)	16(19.8)	0.157
Albuminuria			
Normoalbuminuria	90(55.6)	45(55.6)	
Microalbuminuria	68(42.0)	33(32.7)	0.870
Macroalbuminiuria	4(2.5)	3(3.7)	
Smoking	33(20.5)	20(24.7)	0.234
Alcohol use	86(53.1)	21(25.9)	0.001
Elevated AIP	21(13.0)	6(7.40)	0.279

LDL-C (low density lipoprotein-cholesterol); HDL-C (high density lipoprotein-cholesterol); TC (total cholesterol); TG (triglyceride); AIP(atherogenic index of plasma); GFR (glomerular filtration rate)

The mean serum TC (4.67±0.90 mmol/l vs 4.41±1.01 mmol/l; p = 0.009); TG (1.37±0.40 mmol/l vs 1.25±0.36 mmol/l; p = 0.021), LDL (3.00±0.79 mmol/l vs 2.74±0.90 mmol/l; p = 0.032), blood glucose (6.30±3.13 mmol/l vs 5.76±0.96 mmol/l; p = 0.027) and uric acid (5.59 ±1.79 mg/l vs 3.81 ±1.59; p = <0.001) were significantly higher in the automobile mechanics. The median values of NGAL in the automobile mechanics and controls were 6.04(4.39–6.85) ng/ml and 5.91(4.88–7.24) ng/ml respectively. The median values of TAC in the automobile mechanics and controls were 392.40(228.65–510.55) μmol Trolox equiv/L and 424.50(304.4–592.70) μmol Trolox equiv/L respectively. There was no significant difference in the median values of the TAC and NGAL between the two groups Table 3.

**Table 3:** Comparison of mean values of lipid parameters, NGAL, TAC and uric acid between the automobile mechanics and controls.

Variable	Automobile mechanic Group n = 162	Control Group n = 81	P-value
	Mean±SD /Median (IQR)	Mean (SD)/Median (IQR)	
TC (mmol/l)	4.67±0.90	4.41±1.01	0.009
TG (mmol/l)	1.37±0.40	1.25±0.36	0.021
HDL-C (mmol/l)	1.14±0.24	1.09±0.19	0.107
LDL-C (mmol/l)	3.00±0.79	2.74±0.90	0.032
Blood Glucose (mmol/l)	6.30±3.13	5.76±0.96	0.027
NGAL* (ng/ml)	6.04(4.39–6.85)	5.91(4.88–7.24)	0.199
TAC* (μmol Trolox equiv/L)	392.40(228.65–510.55)	424.50(304.4–592.70)	0.555
Uric Acid (mmol/l)	5.59 ±1.79	3.81 ±1.59	<0.001

* Expressed as Median (IQR)

LDL-C (low density lipoprotein-cholesterol); HDL-C (high density lipoprotein-cholesterol); TC (total cholesterol); TG (triglyceride); AIP(atherogenic index of plasma); TAC (total antioxidant capacity); NGAL (neutrophil gelatinase-associated lipocalin)

Among the automobile mechanics, there was significant positive correlation between serum cadmium, AIP (p = 0.024; r = 0.382); and TG (p = 0.020; r = 0.391). Similarly, there was significant positive correlation between serum lead and NGAL (p = <0.001; r = 0.329) Table 4.

**Table 4:** Correlation between serum heavy metals and some parameters among automobile mechanic.

Parameters		1	2	3	4	5	6	7	8	9	10	11	12
Cadmium	1	1.00											
Lead	2	-0.26	1.00										
TC	3	0.19	-0.08	1.00									
HDL	4	-0.14	0.07	.264**	1.00								
LDL	5	0.15	-0.05	0.942**	0.04	1.00							
TG	6	.391*	-0.15	.319**	0.02	0.13	1.00						
AIP	7	.382*	-0.13	0.09	-.557**	0.07	0.769**	1.00					
NGAL	8	-0.30	.329**	-0.08	0.04	-0.05	-.173*	-.199*	1.00				
TAC	9	0.07	-0.12	-0.06	-.290**	-0.01	0.10	.260**	0.02	1.00			
Creatinine	10	0.09	0.00	-0.06	0.07	-0.11	.160*	0.08	-0.01	0.03	1.00		
GFR	13	-0.06	-0.03	0.07	-0.08	0.14	-.163*	-0.08	-0.02	-0.07	-.906**	1.00	
Uric Acid	14	0.07	-0.11	.330**	0.10	.250**	.341**	.236**	-0.07	-0.08	0.07	-0.01	1.00

*. Correlation is significant at the 0.05 level (2-tailed).

**. Correlation is significant at the 0.01 level (2-tailed).

LDL-C (low density lipoprotein-cholesterol); HDL-C (high density lipoprotein-cholesterol); TC (total cholesterol); TG (triglyceride); AIP(atherogenic index of plasma); TAC (total antioxidant capacity); NGAL (neutrophil gelatinase-associated lipocalin), GFR (glomerular filtration rate)

Among all study participants, there was significant positive correlation between serum cadmium level, AIP (p = 0.016; r = 0.373) and TG (p = 0.004; r = 0.439). There was also positive correlation between serum lead and NGAL levels (p = 0.005; r = 0.206) Table 5.

**Table 5:** Correlation between serum heavy metals and some parameters among automobile mechanic and controls.

Parameters			1	2	3	4	5	6	7	8	9	10	11	12
Cadmium		1	1.00											
Lead		2	-0.29	1.00										
TC		3	0.23	-0.07	1.00									
HDL-C		4	0.02	0.00	.302**	1.00								
LDL-C		5	0.14	-0.02	.949**	0.10	1.00							
TG		6	.439**	-.154*	.334**	0.02	.183**	1.00						
AIP		7	.373*	-0.09	0.09	-.535**	0.09	.781**	1.00					
NGAL		8	-0.28	.206**	-0.10	-0.05	-0.06	-.252**	-.200**	1.00				
TAC		9	0.02	-0.03	0.02	-.228**	0.05	.173**	.284**	0.01	1.00			
Creatinine		10	0.12	0.02	0.00	0.07	-0.05	.171**	0.10	-0.05	0.11	1.00		
GFR		11	-0.10	-0.03	-0.02	-0.10	0.03	-.161*	-0.07	0.00	-.142*	-.905**	1.00	
Uric Acid		12	0.22	-0.12	.310**	0.12	.236**	.314**	.195**	-.167**	-0.05	.158*	-0.10	1.00

*. Correlation is significant at the 0.05 level (2-tailed).

**. Correlation is significant at the 0.01 level (2-tail

LDL-C (low density lipoprotein-cholesterol); HDL-C (high density lipoprotein-cholesterol); TC (total cholesterol); TG (triglyceride); AIP(atherogenic index of plasma); TAC (total antioxidant capacity); NGAL (neutrophil gelatinase-associated lipocalin), GFR (glomerular filtration rate)

## Discussion

This study determined some cardiovascular risk factors, kidney function, and their association with heavy metals among automobile mechanics. Automobile mechanics have notable exposure to heavy metals and a higher prevalence of some cardiovascular risk factors, hence they have higher predisposition to cardiovascular disease and kidney disease.

The mean age of the automobile mechanics in this study was 47 years. This is comparable to 44.6 years reported by Saliu et al [27] in a similar study from Nigeria. The mean age of the study participants was lower than 28.6 years reported in a study done in Nepal [28]. All the participants in this study were males which is similar to report of study by Ozomata et al [29]. This is due to the fact that automobile repair work is a male dominant profession in Nigeria.

The proportion of automobile mechanics that regularly used overall protective coat or wear while working was 50%. This is comparable to 49.3%% reported in Nepal [28], but higher than 40% reported by Ozomata et al [29] in a previous Nigerian study and 27% reported among vehicle repair artisans in a study done in Ghana [30]. Majority of the automobile mechanics in this study sucked petrol directly with their mouth and frequently used petrol to wash their hands to remove oil residue when repairing vehicles. These unhealthy practices especially sucking of petrol with their mouth exposes majority of them to heavy metals directly by ingestion or indirectly by absorption through the oral mucosa in the course of their work. The findings from this study showed the need for automobile mechanics to be regularly sensitized and health educated on the dangerous effects of exposure to heavy metals as well as the need to routinely use protective wears and engage in safe and healthy practices when at work, to limit occupational hazards.

There was significantly higher proportion of automobile mechanics with elevated serum levels of cadmium compared to control. This is similar to the findings from previous studies [31–33]. The harmful occupational practices with consequent excessive exposure to heavy metals may be potentially responsible for the elevated levels. Among the automobile mechanics and the controls in this study, no significant difference was found in the proportion of those with elevated serum lead levels. This finding is different from the reports of similar studies that showed significantly higher serum lead levels in those who were occupationally exposed compared to the control [9, 31, 33]. This finding underscores the need to look beyond occupation as a risk factor for exposure to some heavy metals such as lead. It implies the possible contamination of persons by other potential indirect sources apart from direct contact with petrochemical products. There is need to explore other potential sources of contamination such as soil, water, consumed food items as well as commodities that people are constantly exposed to. This position is strengthened by reports of previous studies that have established higher concentration of heavy metals in soil and water bodies in Nigeria [34–36]. In context, Dix-Cooper et al [37] reported significant association between seafood consumption and the use of herbal remedies with increased serum levels of some heavy metals among Asian women living in Canada. Matouke et al [38] reported high level of heavy metals beyond permissible values in frequently consumed fishes in North Central region of Nigeria. Similarly, Sarkar et al [39] reported higher amount of heavy metal in shrimps from some farms in Bangladesh. Reports from studies by Biose et al [40] and Edogbo et al [35] also showed higher quantities of heavy metals in vegetables, tubers and soil in different parts of Nigeria. The above therefore highlights the need for a holistic approach to ensure the protection of people from both direct and indirect exposure to heavy metals.

Automobile mechanics had significantly higher mean value of total cholesterol and triglyceride compared to the controls in this study. This is similar to the pattern of lipid abnormalities reported in some previous studies among similar study participants [41–43]. There was significant positive association between serum levels of cadmium, triglyceride and AIP in the study participants. This is supported by the finding of the Korean National Environmental Health Survey conducted among 2,519 participants that showed remarkable association between exposure to heavy metals and dyslipidaemia [44]. Similarly, Sharma et al [45] reported significant association between heavy metals and some lipid fractions. Although, studies have reported association between heavy metals and lipid abnormalities, the pathophysiology is not well understood. Overall, the lipid pattern among automobile mechanics suggests that they have higher cardiovascular risk factor compared to the controls. This is similar to findings from previous studies [15, 41–43, 46].

Alcohol consumption was significantly more common among automobile mechanics. About half (53.1%) of them consume alcohol to varying degree. The high prevalence of alcohol use may be due to the fact that artisans such as automobile mechanics habitually consume alcohol based herbal products which are mostly sold or hawked in the their workshops and garages. The frequency of alcohol consumption is similar to 53.4% reported by Akintunde et al. [15]. However, it is higher than 31.2% reported by Ajani et al. [46]. About one-fifth (20.5%) of the automobile mechanics smoked cigarette which is similar to 18.3% reported by Ajani et al, [46] but higher than 10.7% reported by Akintunde et al. [15]. This consumption pattern could predispose them to the adverse cardiovascular and non-cardiovascular consequences of alcohol and tobacco.

The prevalence of hyperuricemia was significantly higher in the automobile mechanics compared to the control. This finding is keeping with report of previous studies conducted within and outside Nigeria [9, 32, 47, 48]. Hyperuricemia is a non-traditional cardiovascular risk factor that is associated with target organ damage, cardiovascular morbidity and mortality [49]. Although the pathophysiology is not fully understood, it has been suggested that uric acid has adverse effect on cardiovascular system by causing endothelial dysfunction, inflammation, increased oxidative stress, insulin resistance, metabolic dysregulation, vasoconstriction and proliferation of vascular smooth muscle [50]. Elevated blood glucose level was also significantly more common in the automobile mechanics. The prevalence rates of these traditional cardiovascular risk factors ranged between 9.9 and 40.7%. The high prevalence of cardiovascular risk observed in this study is similar to previous reports by Akintunde et al. [15].

A higher proportion of automobile mechanics had low GFR, this was not statistically significant. This finding is similar to report of Oktem et al [10] that showed no significant difference in the levels of serum creatinine and GFR between those who were exposed to heavy metals and those who were unexposed. However, it is different from report by Alasia et al [9] that showed significantly lower GFR and higher serum creatinine in those exposed to heavy metals. NGAL is an early marker of kidney injury and has been found to be highly valuable in both acute kidney injury and chronic kidney disease [51]. There was significant positive association between NGAL, cadmium and lead in this study. These findings are supported by the report of Lentini et al [52] that showed that exposure to heavy metals could cause both glomerular and tubular injury in the kidneys.

The limitation of this study was that causal association between heavy metal, cardiovascular risk factor and kidney function could not be ascertained as this was a cross-sectional study. Secondly, we could not exclude the influence of the use supplements in this study. Thirdly, the findings of this study can also not be generalized due to its relatively small study size. The findings of this study can however, serve as a basis for conducting a large population and longitudinal study to ascertain causality between heavy metal exposure and cardiovascular risk and kidney damage.

In conclusion, this study showed that automobile mechanics have a higher prevalence of elevated total cholesterol, elevated blood glucose, alcohol use and hyperuricaemia and exposure to heavy metals such as cadmium and lead. There was positive association between heavy metals, triglyceride, atherogenic index of plasma and NGAL. Regular health education and sensitization should be conducted to educate artisans on the dangers of exposure to heavy metals together with measures to adopt to significantly limit occupational exposures to heavy metals. Policies and environmental laws that would significantly reduce direct and indirect exposure to heavy metals should be properly implemented in Nigeria. Large scale longitudinal study to determine causality between heavy metals, cardiovascular risk and kidney function should be conducted in Nigeria.

## Supporting information

S1 Checklist STROBE statement checklist. (DOCX)

## References

[1] Cardiovascular Diseases. https://www.who.int/health-topics/cardiovascular-diseases#tab=tab_1. Accessed 20th May, 2023.

[2] Roth GA, Mensah GA, Johnson CO, Addolorato G, Ammirati E, Baddour LM et al Global burden of cardiovascular diseases and risk factors, 1990–2019: update from the GBD 2019 study. Journal of the American College of Cardiology 2020:76(25):2982–3021. doi: 10.1016/j.jacc.2020.11.010 33309175 PMC7755038

[3] Gheorghe A, Griffiths U, Murphy A, Legido-Quigley H, Lamptey P, Perel P. The economic burden of cardiovascular disease and hypertension in low-and middle-income countries: a systematic. BMC Public Health 2018;18:975. 10.1186/s.12889-018-5806-x 30081871 PMC6090747

[4] Yusuf S, Joseph P, Rangarajan S, Islam S, Mente A, Hystad P et al. Modifiable risk factors, cardiovascular disease, and mortality in 155 722 individuals from 21 high-income, middle-income, and low-income countries (PURE): a prospective cohort study. The Lancet. 2020;395(10226):795–808. 10.1016/S0140-6736(19)32008-2 PMC8006904 31492503

[5] Utang PB, Eludoyin-Ijekeye CL Impacts of automobile workshops on heavy metals concentrations of urban soils in Obio/Akpor LGA, Rivers State, Nigeria, African Journal of Agric. Research 2013:8(26):3476–3482.

[6] Muze NE, Opara AI, Ibe FC, Njoku OC. Assessment of the geo-environmental effects of activities of auto-mechanic workshops at Alaoji Aba and Elekahia Port Harcourt, Niger Delta, Nigeria. Environ Anal Health Toxicol. 2020;35(2):e2020005. doi: 10.5620/eaht.e2020005 32693557 PMC7374190

[7] Nkwoada AU, Alisa CO, Amakom CM. Pollution in Nigerian auto-mechanic villages: A Review. Environmental Science, Toxicology and Food Technology. 2018;12(7):43–54.

[8] Sönmez F, Dönmez O, Sönmez HM, Keskinoğlu A, Kabasakal C, Mir S. Lead exposure and urinary N-acetyl-D-glucosaminidase activity in adolescent workers in auto repair workshops. J Adolesc Health. 2002;30(3):213–216. 11869929 10.1016/s1054-139x(01)00307-x

[9] Alasia DD, Emem-Chioma PC, Wokoma FS Occupational and environmental lead exposure in Port Harcourt, Nigeria: analysis of its association with renal function indices. Niger J Med 2010;19(4):407–417. doi: 10.4314/njm.v19i4.61965 21526629

[10] Oktem F, Arslan MK, Dündar B, Delibas N, Gültepe M, ErgürhanIlhan. Renal effects and erythrocyte oxidative status in long low level-lead exposed adolescent workers in auto-repair workshops. Arch Toxicol. 2004;78(12):681–77. 15526091 10.1007/s00204-004-0597-5

[11] Oche OM, Nneka OC, Abiola OR, Raji I, Jessica AT, Bala HA, et al Determinants of occupational health hazards among roadside automobile mechanics in Sokoto Metropolis, Nigeria. Ann Afr Med. 2020;19(2):80–88. doi: 10.4103/aam.aam_50_18 32499463 PMC7453945

[12] Akpoveta OV, Osakwe SA. Determination of heavy metal content in refined petroleum products. Journal of Applied Chemistry 2014;7(6):1–2.

[13] Alissa EM, Ferns GA. Heavy metal poisoning and cardiovascular disease. J Toxicol. 2011;2011:870125. doi: 10.1155/2011/870125 21912545 PMC3168898

[14] Charan J, Biswas T. How to calculate sample size for different study designs in medical research? Indian J Psychol Med. 2013;35(2):121–6. doi: 10.4103/0253-7176.116232 24049221 PMC3775042

[15] Akintunde AA, Adeniran J, Akintunde TS, Oloyede TO, Salawu AA, Opadijo OG. Air quality index and cardiovascular risk factors among automobile technicians in Southwest Nigeria. Nigerian Journal of Cardiology. 2019;16(1):32.

[16] Erman A, Rahamimov R, Mashraki T, Levy-Drummer RS, Winkler J, David I, et al. The urine albumin-to-creatinine ratio: assessment of its performance in the renal transplant recipient population. Clin J Am Soc Nephrol.2011;6(4):892–7. doi: 10.2215/CJN.05280610 21212424 PMC3069384

[17] Uddin AH, Khalid RS, Alaama M, Abdualkadar AM, Kasmuri A, Abass SA, et al. Comparative study of three digestion methods for elemental analysis in traditional medicine products using atomic absorption spectrometry. J Anal Sci Technol 2016;7(6). 10.1186/s40543-016-0085-6

[18] Benzie IF, Strain JJ. The ferric reducing ability of plasma (FRAP) as a measure of "antioxidant power": the FRAP assay. Anal Biochem. 1996; 239(1):70–6. doi: 10.1006/abio.1996.0292 8660627

[19] Varshney R, Kale RF. Effect of Calmodulin antagonists on radiation induced lipid peroxidation in Microsomes. Int. J. Rad. Biol., 1990; 58: 733–743. 1977818 10.1080/09553009014552121

[20] Huang PL. A comprehensive definition for metabolic syndrome. Disease models & mechanisms. 2009;2(5–6):231–237. doi: 10.1242/dmm.001180 19407331 PMC2675814

[21] Clinical guidelines on the identification, evaluation, and treatment of overweight and obesity in adults: the Evidence Report. National Institutes of Health. 1998;6(Suppl 2):51–209. 9813653

[22] Expert Panel on Detection, Evaluation, and Treatment of High Blood Cholesterol in Adults. Executive summary of the third report of the national cholesterol education program (NCEP) expert panel on detection, evaluation, and treatment of high blood cholesterol in adults (Adult Treatment Panel III). JAMA. 2001;285:2486–97. doi: 10.1001/jama.285.19.2486 11368702

[23] Levey AS, Eckardt KU, Tsukamoto Y, Levin A, Coresh J, Rossert J et al. Definition and classification of chronic kidney disease: a position statement from Kidney Disease: Improving Global Outcomes (KDIGO). Kid Int. 2005;67(6):2089–2100. doi: 10.1111/j.1523-1755.2005.00365.x 15882252

[24] Hazardous substances database (HSDB). Cadmium. National Library of Medicine Toxicology Data network. 2006.

[25] Centers for Disease Control and Prevention (CDC). Very high blood lead levels among adults—United States, 2002–2011. Morbidity and Mortality Weekly Report 2013;62(47):967–971. 24280917 PMC4585637

[26] Dobiasova M. Atherogenic index of plasma [log(triglycerides/HDL-cholesterol)]: theoretical and practical implications. Clin Chem. 2004;50(7):1113–5. doi: 10.1373/clinchem.2004.033175 15229146

[27] Saliu A, Adebayo O, Kofoworola O, Babatunde O, Ismail A. Comparative assessment of blood lead levels of automobile technicians in organised and roadside garages in Lagos, Nigeria. J Environ Public Health. 2015;2015:976563. doi: 10.1155/2015/976563 25759723 PMC4338385

[28] Khadka R, Pandey I, Gautam L. Occupational health hazards and use of personal protective equipment among auto mechanics in Kathmandu Metropolitan City, Nepal. International Journal of Occupational Safety and Health. 2021;11(1):16–24.

[29] Ozomata EA, Osagiede EF, Onyebujoh TJ. Occupational health hazards and use of personal protective equipment among automobile mechanics in Surulere local government area of Lagos State, Nigeria-a descriptive study. International Journal of Occupational Safety and Health. 2022;12(1):35–44.

[30] Monney I, Bismark DA, Isaac OM, Kuffour RA. Occupational health and safety practices among vehicle repair artisans in an urban area in Ghana. Journal of Environmental and Occupational Health. 2014;3(3):147–53.

[31] Ishola AB, Okechukwu IM, Ashimedua UG, Uchechukwu D, Michael EA, Moses O et al. Serum level of lead, zinc, cadmium, copper and chromium among occupationally exposed automotive workers in Benin city. Int J Environ Pollut Res. 2017;5(1):70–9.

[32] Jung W, Kim Y, Lihm H, Kang J. Associations between blood lead, cadmium, and mercury levels with hyperuricemia in the Korean general population: A retrospective analysis of population-based nationally representative data. Int J Rheum Dis. 2019; 22(8):1435–1444. doi: 10.1111/1756-185X.13632 31215160

[33] Alli LA. Blood level of cadmium and lead in occupationally exposed persons in Gwagwalada, Abuja, Nigeria. Interdiscip Toxicol. 2015;8(3):146–50. doi: 10.1515/intox-2015-0022 27486374 PMC4961911

[34] Opasola OA, Adeolu AT, Iyanda AY, Adewoye SO, Olawale SA. Bioaccumulation of Heavy Metals by Clarias gariepinus (African Catfish) in Asa River, Ilorin, Kwara State. J Health Pollut. 2019;9(21):190303. doi: 10.5696/2156-9614-9.21.190303 30931163 PMC6421952

[35] Edogbo B, Okolocha E, Maikai B, Aluwong T, Uchendu C. Risk analysis of heavy metal contamination in soil, vegetables and fish around Challawa area in Kano State, Nigeria. Scientific African. 2020;7:e00281.

[36] Kolawole TO, Olatunji AS, Jimoh MT, Fajemila OT. Heavy Metal Contamination and Ecological Risk Assessment in Soils and Sediments of an Industrial Area in Southwestern Nigeria. J Health Pollut. 2018;8(19):180906. doi: 10.5696/2156-9614-8.19.180906 30524865 PMC6257164

[37] Dix-Cooper L, Kosatsky T. Blood mercury, lead and cadmium levels and determinants of exposure among newcomer South and East Asian women of reproductive age living in Vancouver, Canada. Science of the Total Environment. 2018;619:1409–19. doi: 10.1016/j.scitotenv.2017.11.126 29734617

[38] Matouke MM, Abdullahi KL. Assessment of heavy metals contamination and human health risk in Clarias gariepinus [Burchell, 1822] collected from Jabi Lake, Abuja, Nigeria. Scientific African. 2020;7:e00292.

[39] Sarkar T, Alam MM, Parvin N, Fardous Z, Chowdhury AZ, Hossain S et al. Assessment of heavy metals contamination and human health risk in shrimp collected from different farms and rivers at Khulna-Satkhira region, Bangladesh. Toxicology reports. 2016;3:346–50. doi: 10.1016/j.toxrep.2016.03.003 28959555 PMC5615835

[40] Biose E, Amaechi CF, Ahator F. Heavy metal contents of some common tubers sold in Benin metropolis, Benin City, Nigeria. FUTY Journal of the Environment. 2020;14(3):84–95.

[41] Festus OO, Ebaluegbeifoh LO, Iyevhobu LO, Dada FL, Iweka FK. Assessment of lipid profile in automobile mechanics in Ekpoma, Edo State. European Journal of Biomedical. 2016;3(9):100–7.

[42] Obi-Ezeani CN, Dioka CE, Meludu SC, Onuora IJ, Usman SO, Onyema-Iloh OB. Blood Pressure and Lipid Profile in Automechanics in Relation to Lead Exposure. Indian J Occup Environ Med. 2019;23(1):28–31. doi: 10.4103/ijoem.IJOEM_122_18 31040586 PMC6477941

[43] Ademuyiwa O, Ugbaja RN, Idumebor F, Adebawo O. Plasma lipid profiles and risk of cardiovascular disease in occupational lead exposure in Abeokuta, Nigeria. Lipids in Health and Disease. 2005;4(1):1–7. doi: 10.1186/1476-511X-4-19 16191200 PMC1253530

[44] Kim DW, Ock J, Moon KW, Park CH. Association between Heavy Metal Exposure and Dyslipidemia among Korean Adults: From the Korean National Environmental Health Survey, 2015–2017. Int J Environ Res Public Health. 2022;19(6):3181. doi: 10.3390/ijerph19063181 35328872 PMC8951064

[45] Sharmar SV, Kumar P, Atam V, Verma A, Murthy RC. Lipid profiles with increased blood lead levels: Risk of cardiovascular disease in battery workers of Lucknow city. J Indian Acad Forensic Med 2012;34:328–31.

[46] Ajani EO, Ajibola A, Salau BA, Odufuwa TK, Odewabi AO. Preliminary report on hepatic and cardiovascular risk assessment of automobile mechanics in Nigeria. African journal of biotechnology. 2011;10(9):1705–11.

[47] Baki AE, Ekiz T, Öztürk GT, Tutkun E, Yilmaz H, Yildizgören MT. The Effects of Lead Exposure on Serum Uric Acid and Hyperuricemia in Young Adult Workers: A Cross-sectional Controlled Study. Arch Rheumatol. 2016;31(1):71–75. doi: 10.5606/ArchRheumatol.2016.5955 29900972 PMC5827869

[48] Adejumo BI, Awelogun KO, Uchuno GA, Emmanuel AM, Dimkpa U, Omosor KI et al. Assessment of renal biomarkers of renal function in commercial automobile workers in Benin City, Edo State, Nigeria. Open Journal of Nephrology. 2018; 8(01):18. doi: 10.4236/ojneph.2018.81003

[49] Muiesan ML, Agabiti-Rosei C, Paini A, Salvetti M. Uric Acid and Cardiovascular Disease: An Update. Eur Cardiol. 2016;11(1):54–59. doi: 10.15420/ecr.2016:4:2 30310447 PMC6159425

[50] Ndrepepa G. Uric acid and cardiovascular disease. Clin Chim Acta. 2018 Sep;484:150–163. doi: 10.1016/j.cca.2018.05.046 29803897

[51] Devarajan P. Neutrophil gelatinase-associated lipocalin (NGAL): a new marker of kidney disease. Scand J Clin Lab Invest Suppl. 2008;241:89–94. doi: 10.1080/00365510802150158 18569973 PMC2528839

[52] Lentini P, Zanoli L, Granata A, Signorelli SS, Castellino P, Dell’Aquila R. Kidney and heavy metals-The role of environmental exposure. Molecular medicine reports. 2017;15(5):3413–9. 28339049 10.3892/mmr.2017.6389

