# Supplementary material for: Cardiovascular risk factors and kidney function among automobile mechanic and their association with serum heavy metals in Southwest Nigeria: A cross-sectional study
Source: PLoS One. 2023 Oct 11;18(10):e0292364. doi: 10.1371/journal.pone.0292364 (PMC10566715; doi:10.1371/journal.pone.0292364)
Supplement: Attachment — Submitted filename: MANUSCRIPT PLOS One.docx [file pone.0292364.s002.docx]

**TITLE:** Cardiovascular Risk Factors and Renal Function among Auto-mechanic and their association with Serum Heavy Metals: [A Cross-sectional Study] This part should be deleted and supplied under material and methods]. However it is helpful to have an idea where the study was carried out !

Oluseyi Ademola Adejumo^1^, Adenike Christianah^1^ Enikuomehin, Adeyemi Ogunleye^2^, Walter Bankole Osungbemiro^3^, Ayodeji Akinwumi Akinbodewa^1^,Olutoyin Morenike Lawal^1^, Stanley Ngoka^4^, Oladimeji Junaid^1^, Kenechuckwu Okonkwo^1^, Emmanuel Oladimeji Alli^1^, Rasheed Olanshile Oloyede^1^

1. Department of Internal Medicine, University of Medical Sciences, Ondo State, Nigeria
2. Department of Medical Laboratory Science, University of Medical Sciences, Ondo State, Nigeria
3. Department of Chemistry, University of Medical Sciences, Ondo State, Nigeria
4. Department of Internal Medicine, Federal University Teaching Hospital, Imo State, Nigeria

**Corresponding author**

Dr Oluseyi Ademola Adejumo

Department of Medicine, University of Medical Sciences,

Ondo State, Nigeria

[oluseyiadejumo2017@gmail.com](mailto:oluseyiadejumo2017@gmail.com)

+2348034225294

Running Title: Cardiovascular Risk Factors and Renal Function among Auto-mechanic and their association with Serum Heavy Metals [I suggest “Cardiovascular risk factors and renal function in auto mechanics in association with serum heavy metals]

**ABSTRACT**

Introduction: The burden of cardiovascular disease (CVD) is huge due to its associated morbidity, mortality and adverse socio-economic impact. Environmental pollution as a risk factor contributes significantly to the burden of CVD especially in the low and middle income countries. One of the effective strategies to reduce CVD burden is to prevent or detect cardiovascular risk factors early in at-risk population. This study determined some cardiovascular risk factors and renal function among auto-mechanic and their association with heavy metals. [among automobile mechanics.] Repitition

Method: This was a cross-sectional study in 162 auto-mechanics and 81 age and sex matched controls. Serum levels of lead, cadmium and some cardiovascular risks were assessed and compared in the two groups. Association between serum lead, cadmium and some cardiovascular risks were determined. [**How? Methods]**

Results: The mean ages of the auto-mechanics and controls were 47.27±9.99 years and 48.94±10.34 years, respectively. Elevated serum cadmium was significantly higher in the mechanics (25.9% vs 7.9%; p=<0.001). The significant cardiovascular risk factors in the auto-mechanics vs controls were elevated total cholesterol (32.1% vs 18.5%; p=0.017), hyperuricemia (20.4% vs 1.2%; p=<0.001), elevated blood glucose (16.0% vs 4.9%’p=0.013); and alcohol use (55.1% vs 30.0%; p=0.001). Among the auto-mechanics, there were significant positive correlation between serum cadmium, atherogenic index of plasma (AIP) (p=0.024; r=0.382) and triglyceride (p=0.020; r=0.391). Significant positive correlation was found between serum lead and neutrophil gelatinase associated lipocalin (NGAL) (p=<0.001; r=0.329). There were significant positive correlation between serum cadmium level, AIP (p=0.016; r=0.373) and TG (p=0.004; r=0.439); between serum lead and NGAL in all the study participants (p=0.005; r=0.206).

Conclusion: Auto-mechanics have notable exposure to heavy metals and a higher prevalence of cardiovascular some risk factors. Health education and sensitisation as well as policies that would regulate exposure of persons to heavy metals should be implemented in Nigeria.

Key word: Auto-mechanic; heavy metal; cardiovascular risk; renal function

**Introduction**

Cardiovascular disease (CVD) is a disease of public health importance due to its increasing prevalence, associated morbidity, mortality and socio-economic impact.^1,2,3^ CVD accounts for 17.9 million deaths globally. The burden of CVD is however higher in low and middle-income countries.^1,2,4^ Cardiovascular risk factors which account for CVD can be either traditional or novel. These risk factors include hypertension, obesity, diabetes mellitus, tobacco use, dyslipidaemia, obesity, depression, environmental pollution, low education, low grip strength and poor diet.^1,4^ The contribution of cardiovascular risk factors as drivers of cardiovascular morbidities and mortality varies in different countries.^1,4^ Prevention or early detection of cardiovascular risk factors constitute one of the major strategies to reduce the burden of CVD. Of the risk factors stated above, environmental pollution and contamination is a more common contributor to CVD in low and middle-income countries compared to high income countries.^1,4^ Occupational practices of artisans such as auto-mechanics may contribute to environmental pollution and contamination.^5-7^ Previous studies within and outside Nigeria have reported higher serum levels of some heavy metals in auto-mechanics than unexposed individuals.^8-10^ Hazardous occupational practices such as regular use of diesel, petrol to wash the hands and feet as well as constant oral sucking of fuel may partly account for higher levels of some heavy metals in automobile mechanics.^11^ The report of Akpoveta et al^12^ specifically showed higher concentration of heavy metals such as zinc and lead in diesel and petrol in Nigeria beyond the generally permissible levels in refined petroleum products.

Although, the pathophysiology of association between heavy metal exposure and CVD is not well understood, both experimental and human studies have implicated the activation of renin-angiotensin-aldosterone system, generation of reactive oxygen species and inflammation.^13^ Heavy metals have also been found to be associated with increased prevalence of some cardiovascular risk factors such hypertension, hyperuricaemia and dyslipidaemia.^13^ [So what is the justification for this study or the gap in knowledge?]

This study determined some cardiovascular risk factors and renal function among auto-mechanic and their association with heavy metals among automobile mechanics. The findings of this study will provide scientific evidence to advocate for safe occupational practices among automobile mechanics. In addition, this study will provide a basis for other studies to establish the association between serum heavy metal, renal function and cardiovascular risks among individuals who have significant occupational exposure.

**METHODOLOGY**

This was a cross-sectional study that was carried out in Ondo State, Nigeria between November 2021 and February, 2022. Ondo state is one of the 36 states in Nigeria and is located in the Southwestern part of the country.

**Study Population**

The study participants comprised of auto-mechanics in Ondo and Akure city, Ondo State. Inclusion criteria were auto-mechanic with a minimum working experience of 1 year, aged ≥ 18 years, and those who gave informed consent. Any auto-mechanic with known renal, heart, and liver diseases was excluded. [ How was this done, Pre or post sampling?] Controls were non-automobile mechanics with no exposure to petrochemicals.

**Sample Size Determination**

The sample size was determined using the formula for single proportion.^14^ The prevalence of a cardiovascular risk factor (generalized obesity) among auto-mechanic used in this calculation was 5.8% based on report from a previous study.^15^ The confidence interval was taken as 95% and the power of the study was 80%. The minimum sample size for this study was 92 after including 10% attrition. A total of 182 auto-mechanics and 91 age and sex matched non-automobile mechanics were consecutively recruited in the study.

**Study Procedure**

Questionnaire was administered to study participants to obtain socio-demographic information; occupational history such as number of years of practice; use of protective overall gown during work; occupational practices relating to the use of petrochemicals; exposure to petrol and diesel; medical history; and social history such as smoking and alcohol use.[ What was the level of education of these participants, was there translation of the questionnaire to local languages?] All the study participants completed the questionnaires which were researcher-administered and underwent physical examination.

Height was measured in metres (m) to the nearest 0.1m using a graduated height scale with the participant in erect position without shoes. Weight was measured in kilogram (kg) to the nearest 0.1kg using standard weighing scale with participants wearing light clothing without shoes, cap or headgear. Body mass index (BMI) was calculated as follow;

BMI (kg/$m^{2}$) = weight (kg)/$\mathrm{height}^{2}$($m^{2}$).

The waist circumference was measured using inelastic tape to the nearest 0.1cm, in the horizontal plane, mid-way between the anterior margin of the lowest rib and the iliac crest with the participant standing comfortably and at the end of normal expiration. Blood pressure was measured using Accosson mercury sphygmomanometer. This was taken with participant comfortably seated with arm rested on a table.

Ten ml of fasting blood samples was collected from the study participants for serum lead, cadmium, fasting serum lipid profile, uric acid, total antioxidant capacity (TAC), neutrophil gelatinase-associated lipocalin (NGAL), creatinine and blood glucose. Five ml of spot urine was collected and analyzed for albumin-creatinine ratio (ACR). ACR was determined using appropriate spectrophotometric methods, [ [Please state or reference method used] Heavy metals levels was determined using atomic spectrometer. [Wrong spelling]

Blood sample preparation and digestion for heavy metal analysis

A modification of the procedure used by Uddin et al^16^ was adopted for the digestion of the blood sample. 3 ml of whole blood sample was placed in a test tube, and a 5 ml mixture of nitric-hydrochloric acid (HNO_3_–HCl) was added to the test tube in a ratio 3:1. The test tube was heated on a hot plate at 100 °C for 3 hours until a clear sample was obtained. After cooling, the sample was then filtered using a 0.45 μm filter paper. The filtrate was made up to 10 mL with deionized water and stored for elemental analysis using an Atomic Absorption Spectrophotometer (Buck 200 model).

TAC was estimated using standard spectrophotometric method based on the ferric reducing antioxidant power (FRAP) assay.^17^ 1.5 mL of freshly constituted solutions of 2,4,6-tripyridyl-s-triazine and Ferric chloride hexahydrate were made to react with 50µL of sample at 37^0^C and the result expressed in µmol Trolox equiv/L. Malondialdehyde (MDA) was measured spectrophotometrically based on its reaction with 2-thiobarturic acid (TBA) in acidic pH, this reaction was measured and MDA value calculated.^18^ Plasma NGAL was analyzed using Sandwich-ELISA kit produced by the Elabscience US.

Definition of terms

Central obesity was defined as waist circumference ≥ 102cm for males.^19^ Generalized obesity was defined as BMI ≥30 kg/m^2^.^20^  Elevated total cholesterol was defined as TC >200 mg/dl; low high-density lipoprotein cholesterol (HDL-C) was defined as HDL-C < 40 mg/dl; elevated low-density lipoprotein cholesterol (LDL-C) was defined as LDL-C >130 mg/dl; and elevated triglyceride (TG) was defined as TG >150 mg/dl.^21^  Normoalbuminuria was defined as ACR value <30mg/g, microalbuminuria was defined as ACR value between 30-299mg/g[ [Usually 300] and macroalbuminuria was defined as ACR value ≥300mg/g.^22^ Renal impairment was defined as a glomerular filtration rate (GFR) of less than 60mls/mins/1.73m^2^.^22^ Elevated serum cadmium was taken as cadmium level above the permissible concentration of 0.03–0.12 μg/dl or 0.0003 - 0.0012 mg/L.^23^ Elevated serum lead was taken as lead level greater than the allowed concentration of 0 - 10 μg/dL or 0 - 0.1 mg/L.^24^ The atherogenic index of plasma (AIP) was defined as the logarithm of the ratio TG and HDL-C (log TG/HDL-C).^25^ AIP value >0.24 was defined as high cardiovascular risk.^25^

Ethical consideration

Informed consent was obtained from all participants and the information was treated with utmost confidentiality. Ethical approval was obtain from the Human Research and Ethics Committee of the University of Medical Sciences, Ondo State.

Statistical Analysis

Data obtained were entered and analyzed using the Statistical Package for Social Sciences (SPSS) version 21.0 software 9 (IBM-SPSS, Armonk, NY: IBM Corporation). Descriptive data were presented as tables and categorical variables of the two groups (auto-mechanics and controls) were expressed as proportions and percentages. Normally distributed data were presented as mean and standard deviation while skewed data ~~was~~ were presented as median and interquartile range. Association between categorical variables was analyzed using Chi-square. Fisher’s exact test was used when the number of counts was less than 5. The level of significance for each test was set at p < 0.05. Correlation was used to determine association between continuous variables.

Results

There were 243 male participants in this study comprising of 162 auto-mechanics and 81 age and sex matched controls. The mean age of the mechanics and the controls were 47.27±9.99 years and 48.94±10.34 years, respectively. Majority of the study participants were between the age group of 40-60 years, Christians, and married. The controls were significantly more educated (p=<0.001) than the auto-mechanics. Ninety-seven (60%) of the auto-mechanics had below 10 years working experience. Table 1

While at work, about 50% of the auto-mechanics regularly wore overall coat, 156 (85.7%) frequently used petrol and diesel to wash their hands and feet and 131 (72.0%) regularly sucked petrol with mouth. Figure1

The prevalence of elevated TC (32.1% vs 18.5%; p=0.017), hyperuricemia (20.4% vs 1.2%; p=<0.001), elevated blood glucose (16.0% vs 4.9%’p=0.013); and alcohol use (55.1% vs 30.0%; p=0.001) were significantly higher in the auto-mechanics compared to the control group. A significantly higher proportion of automobile mechanics had high serum cadmium level compared to the controls (25.3% vs 7.4%; p=<0.001). There was no significant difference in the proportion of auto-mechanics and controls with high serum lead level (82.7% vs 77.8%; p=0.354). Table 2

The mean serum TC (4.67±0.90 mmol/l vs 4.41±1.01 mmol/l; p=0.009); TG (1.37±0.40 mmol/l vs 1.25±0.36 mmol/l; p=0.021), LDL (3.00±0.79 mmol/l vs 2.74±0.90 mmol/l; p=0.032), blood glucose (6.30±3.13 mmol/l vs 5.76±0.96 mmol/l ; p=0.027) and uric acid (5.59 ±1.79 mg/l vs 3.81 ±1.59; p=<0.001) as well as the median NGAL value (6.03(2.47) ng/ml vs 5.68(2.10) ng/ml; p=0.048) were significantly higher in the auto-mechanics. The median value of the TAC was higher among the control although was not significant. Table 3

Among the auto-mechanics, there was significant positive correlation between serum cadmium, AIP (p=0.024; r=0.382); and TG (p=0.020; r=0.391). Similarly, there was significant positive correlation between serum lead and NGAL (p=<0.001; r=0.329).Table 4

Among all study participants, there were significant positive correlation between serum cadmium level, AIP (p=0.016; r=0.373) and TG (p=0.004; r=0.439). There was also positive correlation between serum lead and NGAL levels (p=0.005; r=0.206). Table 5

**Discussion**

This study assessed cardiovascular risk factors and renal function, and their association with some heavy metals among auto-mechanics. The study found that the auto-mechanics had significantly higher prevalence of some of the cardiovascular risk factors as well as elevated serum levels of cadmium compared to the control group who were not auto-mechanics. In addition, a positive association was found between serum levels of some heavy metals, cardiovascular risk factors and renal function. [This is a rehearse of the result again it is not necessary under discussion. What is needed here is the implications of the findings]

The mean age of the automobile mechanics in this study was 47 years. This is comparable to 44.6 years reported by Saliu et al^26^ in a similar study from Nigeria. The mean age of the study participants was lower than 28.6 years reported in a study done in Nepal.^27^ All the participants in this study were males which is similar to report of study by Ozomata et al.^28^ This is due to the fact that automobile repair work is predominantly a male dominant profession in Nigeria.

The proportion of auto-mechanics that regularly used overall protective coat or wear while working was 50%. This is comparable to 49.3%% reported in Nepal^27^, but higher than 40% reported by Ozomata et al^28^ in a previous Nigerian study and 27% reported among vehicle repair artisans in a study done in Ghana.^29^ Majority of the auto-mechanics in this study sucked petrol directly with their mouth and frequently used petrol to wash their hands to remove oil residue when repairing vehicles. These unhealthy practices expose majority of these repairmen to heavy metals in the course of their work. The findings from this study showed the need for auto-mechanics to be regularly sensitized and health educated on the dangerous effects of exposure to heavy metals as well as the need to routinely use protective wears and engage in safe and healthy practices when at work, to limit occupational hazards.

There was significantly higher proportion of auto-mechanics with elevated serum levels of cadmium compared to control. This is similar to the findings from previous studies.^30,31,32^ The harmful occupational practices with consequent excessive exposure to heavy metals potentially responsible for these elevated levels. Among the auto-mechanics and the controls in this study, no significant difference was found in the proportion of those with elevated serum lead levels. This finding is different from the reports of similar studies that showed significantly higher serum lead levels in those who were occupationally exposed compared to the control.^9,30,32^ This finding underscores the need to look beyond occupation as a risk factor for exposure to some heavy metals such as lead. It implies the possible contamination of persons by other potential indirect sources apart from direct contact with petrochemical products. There is need to explore other potential sources of contamination such as soil, water, consumed food items as well as commodities that people are constantly exposed to. This position is strengthened by reports of previous studies that have established higher concentration of heavy metals in soil and water bodies in Nigeria.^33-35^ In context, Dix-Cooper et al^36^ reported significant association between seafood consumption and the use of herbal remedies with increased serum levels of some heavy metals among Asian women living in Canada. Matouke et al^37^ reported high level of heavy metals beyond permissible values in frequently consumed fishes in North Central region of Nigeria. Similarly, Sarkar et al^38^ reported higher amount of heavy metal in shrimps from some farms in Bangladesh. Reports from studies by Biose et al^39^ and Edogbo et al^40^ also showed higher quantities of heavy metals in vegetables, tubers and soil in different parts of Nigeria. The above therefore highlights the need for a holistic approach to ensure the protection of people from both direct and indirect exposure to heavy metals.

Auto-mechanics had significantly higher mean value of total cholesterol and triglyceride compared to the controls in this study. This is similar to the pattern of lipid abnormalities reported in previous studies among similar study participants.^42,43^ There was however no significant difference in the mean value of HDL-C between the automobile mechanics and the controls. This is similar to findings of studies done by Ademuyiwa et al.^43^ and Festus et al.^41^ There was significant positive association between serum levels of cadmium, triglyceride and AIP in the study participants. This is supported by the finding of the Korean National Environmental Health Survey conducted among 2,519 participants which showed remarkable association between exposure to heavy metals and dyslipidaemia.^44^ Similarly, Sharma et al^45^ reported significant association between heavy metals and some lipid fractions. Although, studies have reported association between heavy metals and lipid abnormalities, the pathophysiology is not well understood. Similar to the study by Igharo et al,^46^ AIP was not found to be significantly higher in auto-mechanics compared to the controls in this study . However, this finding is at variance with the study report by Ajani et al.^47^ Overall, the lipid pattern among auto-mechanics suggests that they have higher cardiovascular risk factor compared to the controls. This is similar to findings from previous studies.^15,41,42,43,47^

Alcohol consumption was significantly more common among auto-mechanics. About 55% of them consume alcohol to varying degree. The high prevalence of alcohol use may be due to the fact that artisans such as auto-mechanics habitually consume alcohol based herbal products which are mostly sold or hawked in the automobile workshops and garages. The frequency of alcohol consumption is similar to 53.4% reported by Akintunde et al.^15^ However, it is higher than 31.2% reported by Ajani et al.^47^ There was no significant difference in the proportion of the auto-mechanics that smoked cigarette compared to the control. About 21% of them smoked cigarette which is similar to 18.3% reported by Ajani et al,^47^ but higher than 10.7% reported by Akintunde et al.^15^ This consumption pattern could predispose them to the adverse cardiovascular and non-cardiovascular consequences of alcohol and tobacco.

There was no significant difference in the proportion of those with common traditional cardiovascular risk factors such as hypertension, generalized and abdominal obesity between the two groups of study participants. Elevated blood glucose level was however significantly more common in the auto- mechanics. The prevalence rates of these traditional cardiovascular risk factors ranged between 9.9 and 40.7%. The high prevalence of cardiovascular risk observed in this study is similar to previous reports by Akintunde et al.^15^

The prevalence of hyperuricemia was significantly higher in the auto-mechanics compared to the control. This finding is keeping with report of previous studies conducted within and outside Nigeria.^9,31,48,49^ Hyperuricemia is a non-traditional cardiovascular risk factor that is associated with target organ damage, cardiovascular morbidity and mortality.^50^ Although the pathophysiology is fully understood, it has been suggested that uric acid has adverse effect on cardiovascular system by causing endothelial dysfunction, inflammation, increased oxidative stress, insulin resistance, metabolic dysregulation, vasoconstriction and proliferation of vascular smooth muscle.^51^

There was no significant difference in the mean serum creatinine and GFR of the auto-mechanics and control in this study. Although, a higher proportion of automobile mechanics had lower GFR, this was not statistically significant. This finding is similar to report of Oktem et al^10^ that showed no significant difference in the levels of serum creatinine and GFR between those who were exposed to heavy metals and those who were unexposed. However, it is different from report by Alasia et al^9^ that showed significantly lower GFR and higher serum creatinine in those exposed to heavy metals

NGAL is an early marker of kidney injury and has been found to be highly valuable in both acute kidney injury and chronic kidney disease.^52^ Serum NGAL level was found to be significantly higher in the auto-mechanics compared with the control. This finding supports report by Oktem et al^10^ that showed significantly higher level of N-acetyl-beta-D-glucosaminidase, an early marker of kidney injury in those exposed to heavy metal. There are evidences that showed that exposure to heavy metals could cause both glomerular and tubular injury in the kidneys.^53^

The limitation of this study was that causal association between heavy metal, cardiovascular risk factor and renal function could not be ascertained as this was a cross-sectional study. The findings of this study can also not be generalized due to its relatively small study size. The findings of this study can however serve as a basis for conducting a large population and longitudinal study to ascertain causality between heavy metal exposure and cardiovascular risk and kidney damage.

In conclusion, this study showed that auto-mechanics have a higher prevalence of some cardiovascular risk factors and exposure to heavy metals. There was an association between serum lead and cadmium levels and some cardiovascular risk factors as well as markers of kidney damage. Regular health education and sensitization should be conducted to educate artisans on the dangers of exposure to heavy metals together with measures to adopt to significantly limit occupational exposures to heavy metals. Policies and environmental laws that would significantly reduce direct and indirect exposure to heavy metals should be properly implemented in Nigeria. Large scale longitudinal study to determine causality between heavy metals, cardiovascular risk and renal function should be conducted in Nigeria.

References

1. Cardiovascular Diseases. <https://www.who.int/health-topics/cardiovascular-diseases#tab=tab_1>. Accessed 20th May, 2023
2. Roth GA, Mensah GA, Johnson CO, Addolorato G, Ammirati E, Baddour LM et al Global burden of cardiovascular diseases and risk factors, 1990–2019: update from the GBD 2019 study. Journal of the American College of Cardiology 2020:76(25):2982-3021.
3. Gheorghe A, Griffiths U, Murphy A, Legido-Quigley H, Lamptey P, Perel P. The economic burden of cardiovascular disease and hypertension in low-and middle-income countries: a systematic. . BMC Public Health 2018;18:975 https://doi. org/10.1186/s 12889-018-5806-x
4. Yusuf S, Joseph P, Rangarajan S, Islam S, Mente A, Hystad P et al. Modifiable risk factors, cardiovascular disease, and mortality in 155 722 individuals from 21 high-income, middle-income, and low-income countries (PURE): a prospective cohort study. The Lancet. 2020;395(10226):795-808.
5. Utang PB, Eludoyin-Ijekeye CL Impacts of automobile workshops on heavy metals concentrations of urban soils in Obio/Akpor LGA, Rivers State, Nigeria, African Journal of Agric. Research 2013:8(26):3476-3482
6. Muze NE, Opara AI, Ibe FC, Njoku OC. Assessment of the geo-environmental effects of activities of auto-mechanic workshops at Alaoji Aba and Elekahia Port Harcourt, Niger Delta, Nigeria. Environ Anal Health Toxicol. 2020;35(2):e2020005. doi: 10.5620/eaht.e2020005.
7. Nkwoada AU, Alisa CO, Amakom CM. Pollution in Nigerian auto-mechanic villages: A Review. Environmental Science, Toxicology and Food Technology. 2018;12(7):43-54.
8. Sönmez F, Dönmez O, Sönmez HM, Keskinog˘lu A, Kabasakal C, Mir S. Lead exposure and urinary N-acetyl-D-glucosaminidase activity in adolescent workers in auto repair workshops. J Adolesc Health. 2002;30(3):213-216
9. Alasia DD, Emem-Chioma PC, Wokoma FS Occupational and environmental lead exposure in Port Harcourt, Nigeria: analysis of its association with renal function indices. Niger J Med 2010;19(4):407-417
10. [Oktem F](https://www.ncbi.nlm.nih.gov/pubmed/?term=Oktem%20F%5BAuthor%5D&cauthor=true&cauthor_uid=15526091), [Arslan MK](https://www.ncbi.nlm.nih.gov/pubmed/?term=Arslan%20MK%5BAuthor%5D&cauthor=true&cauthor_uid=15526091), [Dündar B](https://www.ncbi.nlm.nih.gov/pubmed/?term=D%C3%BCndar%20B%5BAuthor%5D&cauthor=true&cauthor_uid=15526091), [Delibas N](https://www.ncbi.nlm.nih.gov/pubmed/?term=Delibas%20N%5BAuthor%5D&cauthor=true&cauthor_uid=15526091), [Gültepe M](https://www.ncbi.nlm.nih.gov/pubmed/?term=G%C3%BCltepe%20M%5BAuthor%5D&cauthor=true&cauthor_uid=15526091), [ErgürhanIlhan](https://www.ncbi.nlm.nih.gov/pubmed/?term=Erg%C3%BCrhan%20Ilhan%20I%5BAuthor%5D&cauthor=true&cauthor_uid=15526091) . Renal effects and erythrocyte oxidative status in long low level-lead exposed adolescent workers in auto-repair workshops. [Arch Toxicol.](https://www.ncbi.nlm.nih.gov/pubmed/15526091) 2004;78(12):681-77.
11. Oche OM, Nneka OC, Abiola OR, Raji I, Jessica AT, Bala HA et al Determinants of occupational health hazards among roadside automobile mechanics in Sokoto Metropolis, Nigeria. Ann Afr Med. 2020;19(2):80-88.
12. Akpoveta OV, Osakwe SA. Determination of heavy metal content in refined petroleum products. Journal of Applied Chemistry 2014;7(6):1-2
13. Alissa EM, Ferns GA. Heavy metal poisoning and cardiovascular disease. J Toxicol. 2011;2011:870125. doi: 10.1155/2011/870125.
14. Charan J, Biswas T. How to calculate sample size for different study designs in medical research? Indian J Psychol Med. 2013;35(2):121-6.
15. Akintunde AA, Adeniran J, Akintunde TS, Oloyede TO, Salawu AA, Opadijo OG. Air quality index and cardiovascular risk factors among automobile technicians in Southwest Nigeria. Nigerian Journal of Cardiology. 2019;16(1):32
16. Uddin AH, Khalid RS, Alaama M, Abdualkadar AM, Kasmuri A, Abass SA et al. Comparative study of three digestion methods for elemental analysis in traditional medicine products using atomic absorption spectrometry. J Anal Sci Technol 2016;7(6). <https://doi.org/10.1186/s40543-016-0085-6>
17. Benzie IF, Strain JJ. The ferric reducing ability of plasma (FRAP) as a measure of "antioxidant power": the FRAP assay. Anal Biochem. 1996; 239(1):70-6
18. Varshney R, Kale RF. Effect of Calmodulin antagonists on radiation induced lipid peroxidation in Microsomes. Int. J. Rad. Biol., 1990; 58: 733- 743.
19. Huang PL. A comprehensive definition for metabolic syndrome. Disease models & mechanisms. 2009;2(5-6):231-237.
20. Clinical guidelines on the identification, evaluation, and treatment of overweight and obesity in adults: the Evidence Report. National Institutes of Health. 1998;6(Suppl 2):51-209.
21. Expert Panel on Detection, Evaluation, and Treatment of High Blood Cholesterol in Adults. Executive summary of the third report of the national cholesterol education program (NCEP) expert panel on detection, evaluation, and treatment of high blood cholesterol in adults (Adult Treatment Panel III). JAMA. 2001;285:2486-97.
22. Levey AS, Eckardt KU, Tsukamoto Y, Levin A, Coresh J, Rossert J et al. Definition and classification of chronic kidney disease: a position statement from Kidney Disease: Improving Global Outcomes (KDIGO). Kid Int. 2005;67(6):2089-2100
23. Hazardous substances database (HSDB). Cadmium. National Library of Medicine Toxicology Data network. 2006
24. Centers for Disease Control and Prevention (CDC). Very high blood lead levels among adults - United States, 2002-2011. Morbidity and Mortality Weekly Report 2013;62(47):967–971
25. Dobiasova M. Atherogenic index of plasma [log(triglycerides/HDL-cholesterol)]: theoretical and practical implications. Clin Chem. 2004;50(7):1113–5
26. Saliu A, Adebayo O, Kofoworola O, Babatunde O, Ismail A. Comparative assessment of blood lead levels of automobile technicians in organised and roadside garages in Lagos, Nigeria. J Environ Public Health. 2015;2015:976563. doi: 10.1155/2015/976563.
27. Khadka R, Pandey I, Gautam L. Occupational health hazards and use of personal protective equipment among auto mechanics in Kathmandu Metropolitan City, Nepal. International Journal of Occupational Safety and Health. 2021;11(1):16-24.
28. Ozomata EA, Osagiede EF, Onyebujoh TJ. Occupational health hazards and use of personal protective equipment among automobile mechanics in Surulere local government area of Lagos State, Nigeria-a descriptive study. International Journal of Occupational Safety and Health. 2022;12(1):35-44.
29. Monney I, Bismark DA, Isaac OM, Kuffour RA. Occupational health and safety practices among vehicle repair artisans in an urban area in Ghana. Journal of Environmental and Occupational Health. 2014;3(3):147-53.
30. Ishola AB, Okechukwu IM, Ashimedua UG, Uchechukwu D, Michael EA, Moses O et al. Serum level of lead, zinc, cadmium, copper and chromium among occupationally exposed automotive workers in Benin city. Int J Environ Pollut Res. 2017;5(1):70-9.
31. Jung W, Kim Y, Lihm H, Kang J. Associations between blood lead, cadmium, and mercury levels with hyperuricemia in the Korean general population: A retrospective analysis of population-based nationally representative data. Int J Rheum Dis. 2019 ;22(8):1435-1444.
32. Alli LA. Blood level of cadmium and lead in occupationally exposed persons in Gwagwalada, Abuja, Nigeria. Interdiscip Toxicol. 2015;8(3):146-50
33. Opasola OA, Adeolu AT, Iyanda AY, Adewoye SO, Olawale SA. Bioaccumulation of Heavy Metals by Clarias gariepinus (African Catfish) in Asa River, Ilorin, Kwara State. J Health Pollut. 2019;9(21):190303. doi: 10.5696/2156-9614-9.21.190303.
34. Edogbo B, Okolocha E, Maikai B, Aluwong T, Uchendu C. Risk analysis of heavy metal contamination in soil, vegetables and fish around Challawa area in Kano State, Nigeria. Scientific African. 2020;7:e00281.
35. Kolawole TO, Olatunji AS, Jimoh MT, Fajemila OT. Heavy Metal Contamination and Ecological Risk Assessment in Soils and Sediments of an Industrial Area in Southwestern Nigeria. J Health Pollut. 2018;8(19):180906. doi: 10.5696/2156-9614-8.19.180906.
36. Dix-Cooper L, Kosatsky T. Blood mercury, lead and cadmium levels and determinants of exposure among newcomer South and East Asian women of reproductive age living in Vancouver, Canada. Science of the Total Environment. 2018;619:1409-19.
37. Matouke MM, Abdullahi KL. Assessment of heavy metals contamination and human health risk in Clarias gariepinus [Burchell, 1822] collected from Jabi Lake, Abuja, Nigeria. Scientific African. 2020;7:e00292.
38. Sarkar T, Alam MM, Parvin N, Fardous Z, Chowdhury AZ, Hossain S et al. Assessment of heavy metals contamination and human health risk in shrimp collected from different farms and rivers at Khulna-Satkhira region, Bangladesh. Toxicology reports. 2016;3:346-50.
39. Biose E, Amaechi CF, Ahator F. Heavy metal contents of some common tubers sold in Benin metropolis, Benin City, Nigeria. FUTY Journal of the Environment. 2020;14(3):84-95.
40. Edogbo B, Okolocha E, Maikai B, Aluwong T, Uchendu C. Risk analysis of heavy metal contamination in soil, vegetables and fish around Challawa area in Kano State, Nigeria. Scientific African. 2020;7:e00281.
41. Festus OO, Ebaluegbeifoh LO, Iyevhobu LO, Dada FL, Iweka FK. Assessment of lipid profile in automobile mechanics in Ekpoma, Edo State. European Journal of Biomedical. 2016;3(9):100-7.
42. Obi-Ezeani CN, Dioka CE, Meludu SC, Onuora IJ, Usman SO, Onyema-Iloh OB. Blood Pressure and Lipid Profile in Automechanics in Relation to Lead Exposure. Indian J Occup Environ Med. 2019;23(1):28-31
43. Ademuyiwa O, Ugbaja RN, Idumebor F, Adebawo O. Plasma lipid profiles and risk of cardiovascular disease in occupational lead exposure in Abeokuta, Nigeria. Lipids in Health and Disease. 2005;4(1):1-7.
44. Kim DW, Ock J, Moon KW, Park CH. Association between Heavy Metal Exposure and Dyslipidemia among Korean Adults: From the Korean National Environmental Health Survey, 2015-2017. Int J Environ Res Public Health. 2022;19(6):3181. doi: 10.3390/ijerph19063181
45. Sharmar SV, Kumar P, Atam V, Verma A, Murthy RC. Lipid profiles with increased blood lead levels: Risk of cardiovascular disease in battery workers of Lucknow city. J Indian Acad Forensic Med 2012;34:328-31.
46. Igharo OG, Akinfenwa Y, Isara AR, Idomeh FA, Nwobi NL, Anetor JI et al Lipid profile and Atherogenic Indices in Nigerians Occupationally Exposed to e-waste: A Cardiovascular Risk Assessment Study. Maedica (Bucur). 2020;15(2):196-205.
47. Ajani EO, Ajibola A, Salau BA, Odufuwa TK, Odewabi AO. Preliminary report on hepatic and cardiovascular risk assessment of automobile mechanics in Nigeria. African journal of biotechnology. 2011;10(9):1705-11.
48. Baki AE, Ekiz T, Öztürk GT, Tutkun E, Yilmaz H, Yildizgören MT. The Effects of Lead Exposure on Serum Uric Acid and Hyperuricemia in Young Adult Workers: A Cross-sectional Controlled Study. Arch Rheumatol. 2016;31(1):71-75.
49. Adejumo BI, Awelogun KO, Uchuno GA, Emmanuel AM, Dimkpa U, Omosor KI et al. Assessment of renal biomarkers of renal function in commercial automobile workers in Benin City, Edo State, Nigeria. Open Journal of Nephrology. 2018 ;8(01):18.doi. [10.4236/ojneph.2018.81003](https://doi.org/10.4236/ojneph.2018.81003)
50. Muiesan ML, Agabiti-Rosei C, Paini A, Salvetti M. Uric Acid and Cardiovascular Disease: An Update. Eur Cardiol. 2016;11(1):54-59
51. Ndrepepa G. Uric acid and cardiovascular disease. Clin Chim Acta. 2018 Sep;484:150-163
52. Devarajan P. Neutrophil gelatinase-associated lipocalin (NGAL): a new marker of kidney disease. Scand J Clin Lab Invest Suppl. 2008;241:89-94.
53. Lentini P, Zanoli L, Granata A, Signorelli SS, Castellino P, Dell'Aquila R. Kidney and heavy metals-The role of environmental exposure. Molecular medicine reports. 2017;15(5):3413-9.

Authors’ contributions

OAA, ACE, AO, WBO, OML, AAA, AJ and SN were involved in conceptualization of the study. All authors were involved in literature review, design of methodology and data collection. WBO, OAA, AO and OML were involved in data analysis. OAA, ACE, AO, WBO, OML, AAA, AJ and SN were involved data interpretation. OAA, ACE, ROO, EOA, OJ were involved in writing original draft. OJ, OML and OAA were involved in the manuscript review and editing. All authors read and approved the final draft.

Data Availability statement

The datasets generated during and/or analyzed during the current study are available from the corresponding author on reasonable request.

Declaration of interests

The authors have no conflicts of interest to declare

Table 1: Socio-demographic Characteristics of Study Participants

| Variables | Auto-mechanic  n= 162  Frequency (%) | Control Group  n =81  Frequency (%) | p-value |
| --- | --- | --- | --- |
| Age |  |  |  |
| Mean Age | 47.27±9.99 | 48.94±10.34 |  |
| <40 years | 27(16.7) | 13(16.0) | 0.992 |
| 40-60 years | 113(69.8) | 57(70.4) |  |
| ≥61 years | 22(13.5) | 11(13.6) |  |
|  |  |  |  |
| Gender |  |  |  |
| Male | 162(100)) | 81(100) |  |
|  |  |  |  |
| Marital Status |  |  |  |
| Single | 10(6.3) | 5(7.1) | 0.946 |
| Married | 145(91.8) | 64(91.4) |  |
| Divorced | 3(1.9) | 1(1.4) |  |
|  |  |  |  |
| Religion |  |  |  |
| Christianity | 111(69.8) | 69(98.6) | <0.001 |
| Islam | 47(29.6) | 1(1.4) |  |
| Others | 1(0.6) | 0(0) |  |
|  |  |  |  |
| Educational Level |  |  |  |
| None | 3(1.9) | 1(1.4) | <0.001 |
| Primary | 54(34.0) | 5(7.1) |  |
| Secondary | 90(56.6) | 18(25.7) |  |
| Tertiary | 12(7.5) | 46(65.7) |  |
|  |  |  |  |
| Working Experience |  |  |  |
| <10 years | 97(59.9) |  |  |
| 10-20 years | 38(23.5) |  |  |
| >20 years | 27(16.7) |  |  |

Table 2: Comparison of Cardiovascular Risk between the Auto-mechanics and Controls

|  | Auto-mechanic Group  Frequency (%) | Control Group  Frequency (%) | P-value |
| --- | --- | --- | --- |
| Elevated Serum Cadmium | 41(25.3) | 6(7.4) | 0.002 |
| Elevated Serum Lead | 134(82.7) | 63(77.8) | 0.363 |
| High LDL-C | 52(32.1) | 19(23.5) | 0.105 |
| Low HDL-C | 57(35.2) | 31(38.3) | 0.369 |
| High TC | 52(32.1) | 15(18.5) | 0.017 |
| High TG | 30(18.5) | 8(9.9) | 0.093 |
| Hyperuricemia | 33(20.4) | 1(1.2) | <0.001 |
| Elevated Blood Glucose | 26(16.1) | 4(4.9) | 0.013 |
| Hypertension | 57(35.2) | 39(48.2) | 0.070 |
| Obesity/Overweight | 66(40.7) | 33(40.7) | 1.00 |
| Abdominal obesity | 16(9.9) | 8(9.9) | 1.00 |
| Reduce GFR | 43(26.5) | 16(19.8) | 0.157 |
| Albuminuria  Normoalbuminuria  Microalbuminuria  Macroalbuminiuria | 90(55.6)  68(42.0)  4(2.5) | 45(55.6)  33(32.7)  3(3.7) | 0.870 |
| Smoking | 33(20.5) | 20(24.7) | 0.234 |
| Alcohol use | 86(53.1) | 21(25.9) | 0.001 |
| Elevated AIP | 21(13.0) | 6(7.40) | 0.279 |

Table 3: Comparison of Mean Values of Lipid Parameters, NGAL, TAC and Uric Acid between the Auto-mechanics and Controls

| Variable | Auto-mechanic Group  N=162  Mean±SD /Median (IQR) | Non-mechanic Group  N=81  Mean (SD)/Median (IQR) | P-value |
| --- | --- | --- | --- |
|  |  |  |  |
| Serum TC (mmol/l) | 4.67±0.90 | 4.41±1.01 | 0.009 |
| Serum TG (mmol/l) | 1.37±0.40 | 1.25±0.36 | 0.021 |
| Serum HDL-C (mmol/l) | 1.14±0.24 | 1.09±0.19 | 0.107 |
| Serum LDL-C (mmol/l) | 3.00±0.79 | 2.74±0.90 | 0.032 |
| Serum Blood Glucose (mmol/l) | 6.30±3.13 | 5.76±0.96 | 0.027 |
| Serum NGAL* (ng/ml) | 6.03(2.47) | 5.68(2.10) | 0.048 |
| Serum TAC* (in µmol Trolox equiv/L. | 392.40(221.90) | 424.50(288.3) | 0.555 |
| Serum Uric Acid (mmol/l) | 5.59 ±1.79 | 3.81 ±1.59 | <0.001 |

* Expressed as Median (IQR)

Table 4: Correlation between Serum Heavy Metals and Some Parameters among Auto-mechanic

| Parameters | | 1 | 2 | 3 | 4 | 5 | 6 | 7 | 8 | 9 | 10 | 11 | 12 |
| --- | --- | --- | --- | --- | --- | --- | --- | --- | --- | --- | --- | --- | --- |
| Cadmium | 1 | 1.00 |  |  |  |  |  |  |  |  |  |  |  |
| Lead | 2 | -0.26 | 1.00 |  |  |  |  |  |  |  |  |  |  |
| TC | 3 | 0.19 | -0.08 | 1.00 |  |  |  |  |  |  |  |  |  |
| HDL | 4 | -0.14 | 0.07 | .264^**^ | 1.00 |  |  |  |  |  |  |  |  |
| LDL | 5 | 0.15 | -0.05 | 0.942^**^ | 0.04 | 1.00 |  |  |  |  |  |  |  |
| TG | 6 | .391^*^ | -0.15 | .319^**^ | 0.02 | 0.13 | 1.00 |  |  |  |  |  |  |
| AIP | 7 | .382^*^ | -0.13 | 0.09 | -.557^**^ | 0.07 | 0.769^**^ | 1.00 |  |  |  |  |  |
| NGAL | 8 | -0.30 | .329^**^ | -0.08 | 0.04 | -0.05 | -.173^*^ | -.199^*^ | 1.00 |  |  |  |  |
| TAC | 9 | 0.07 | -0.12 | -0.06 | -.290^**^ | -0.01 | 0.10 | .260^**^ | 0.02 | 1.00 |  |  |  |
| CR | 10 | 0.09 | 0.00 | -0.06 | 0.07 | -0.11 | .160^*^ | 0.08 | -0.01 | 0.03 | 1.00 |  |  |
| GFR | 13 | -0.06 | -0.03 | 0.07 | -0.08 | 0.14 | -.163^*^ | -0.08 | -0.02 | -0.07 | -.906^**^ | 1.00 |  |
| Uric Acid | 14 | 0.07 | -0.11 | .330^**^ | 0.10 | .250^**^ | .341^**^ | .236^**^ | -0.07 | -0.08 | 0.07 | -0.01 | 1.00 |

*. Correlation is significant at the 0.05 level (2-tailed).

**. Correlation is significant at the 0.01 level (2-tailed).

~~u~~Table 5: Correlation between Serum Heavy Metals and Some Parameters Among Auto-mechanic and Controls

| Parameters |  | | 1 | 2 | 3 | 4 | 5 | 6 | 7 | 8 | 9 | 10 | 11 | 12 |
| --- | --- | --- | --- | --- | --- | --- | --- | --- | --- | --- | --- | --- | --- | --- |
| Cadmium | | 1 | 1.00 |  |  |  |  |  |  |  |  |  |  |  |
| Lead | | 2 | -0.29 | 1.00 |  |  |  |  |  |  |  |  |  |  |
| TC | | 3 | 0.23 | -0.07 | 1.00 |  |  |  |  |  |  |  |  |  |
| HDL-C | | 4 | 0.02 | 0.00 | .302^**^ | 1.00 |  |  |  |  |  |  |  |  |
| LDL-C | | 5 | 0.14 | -0.02 | .949^**^ | 0.10 | 1.00 |  |  |  |  |  |  |  |
| TG | | 6 | .439^**^ | -.154^*^ | .334^**^ | 0.02 | .183^**^ | 1.00 |  |  |  |  |  |  |
| AIP | | 7 | .373^*^ | -0.09 | 0.09 | -.535^**^ | 0.09 | .781^**^ | 1.00 |  |  |  |  |  |
| NGAL | | 8 | -0.28 | .206^**^ | -0.10 | -0.05 | -0.06 | -.252^**^ | -.200^**^ | 1.00 |  |  |  |  |
| TAC | | 9 | 0.02 | -0.03 | 0.02 | -.228^**^ | 0.05 | .173^**^ | .284^**^ | 0.01 | 1.00 |  |  |  |
| CR | | 10 | 0.12 | 0.02 | 0.00 | 0.07 | -0.05 | .171^**^ | 0.10 | -0.05 | 0.11 | 1.00 |  |  |
| GFR | | 11 | -0.10 | -0.03 | -0.02 | -0.10 | 0.03 | -.161^*^ | -0.07 | 0.00 | -.142^*^ | -.905^**^ | 1.00 |  |
| Uric Acid | | 12 | 0.22 | -0.12 | .310^**^ | 0.12 | .236^**^ | .314^**^ | .195^**^ | -.167^**^ | -0.05 | .158^*^ | -0.10 | 1.00 |

*. Correlation is significant at the 0.05 level (2-tailed).

**. Correlation is significant at the 0.01 level (2-tailed)
