# Supplementary material for: Cardiovascular risk factors and kidney function among automobile mechanic and their association with serum heavy metals in Southwest Nigeria: A cross-sectional study
Source: PLoS One. 2023 Oct 11;18(10):e0292364. doi: 10.1371/journal.pone.0292364 (PMC10566715; doi:10.1371/journal.pone.0292364)
Supplement: Attachment — Submitted filename: Reviewer report on Manuscript.docx [file pone.0292364.s003.docx]

Reviewer report on Manuscript.**: Titled** Cardiovascular Risk Factors and Renal Function among Auto-mechanic and their association with Serum Heavy Metals: []

Tittle: This phrase “A Cross-sectional Study” should be deleted and supplied under material and methods.. However, it is helpful to have an idea where the study was carried out as part of the title.

**Lines 22-24**. For the short running title, I suggest “Cardiovascular risk factors and renal function in auto mechanics in association with serum heavy metals.

In Abstract Line 39, There was a repetition in the sentence which should be corrected.

Introduction. Line 88. The authors did not clearly justify the research gap for this study. This should be highlighted in the introduction.

Lines 102 -103. Study population I would like to know how this was achieved i.e. the statement “Any auto-mechanic with known renal, heart, and liver diseases was excluded". [ How was this done? Pre or post sampling?]

How did we exclude the use of supplements like herbal teas that enables detoxifications from being responsible for the results we are seeing.

It would also be interesting to look at the lead and cadmium levels according to years of experience of the auto mechanics, an interesting result may come out here.

Lines131-132. Please state or reference method used for ACR .

Results Table 3. The repetition of the word serum should be removed having been initially stated in the methodology section.

Discussion. Lines 203-206 This is a rehearse of the results again it is not necessary under discussion. What is needed here is the implications of the findings. Please comply with this.

The discussion was lengthy on what was not present in this study among the automobile workers. The oral sucking of petrol presupposes ingestion from oral mucosa which could lead to elevated cadmium of lead. This should have been emphasised.

**Conclusion**

The study is informative and adds to the body of knowledge. It is acceptable after these minor corrections.
