# Supplementary material for: Cardiovascular risk factors and kidney function among automobile mechanic and their association with serum heavy metals in Southwest Nigeria: A cross-sectional study
Source: PLoS One. 2023 Oct 11;18(10):e0292364. doi: 10.1371/journal.pone.0292364 (PMC10566715; doi:10.1371/journal.pone.0292364)
Supplement: Attachment — Submitted filename: PLOS ONE REVIEW.docx [file pone.0292364.s004.docx]

Dear Editor-in-Chief,

Thank you for the priviledge given to review the manuscript: Cardiovascular Risk Factors and Renal Function among Auto-mechanic and their association with Serum Heavy Metals: A Cross-sectional Study.

Adejumo and colleagues have endeavored to evaluate the possible relationship(s) between serum heavy metals (lead and cadmium) and cardiovascular risk factors, and renal function among auto-mechanic which is a worthwhile adventure.

Below are my observations, comments and suggestions:

Abstract:

Introduction:

The sentence: This study determined some cardiovascular risk factors and renal function among auto-mechanic and their association with heavy metals among automobile mechanics.

It may not be necessary to repeat the word auto-mechanic (i.e automobile: mechanic). Thus, the sentence could be simply put as:

This study determined some cardiovascular risk factors, renal function, and their association with heavy metals among automobile mechanics.

OR:

This study determined some cardiovascular risk factors and renal function among auto-mechanic and their association with heavy metals

Method:

Associations between serum lead, cadmium and some cardiovascular risks were determined.

Results

There were significant positive correlations between serum cadmium level.

Conclusion:

Auto-mechanics have notable exposure to heavy metals and they have a higher prevalence of cardiovascular some risk factors.

Auto-mechanics have notable exposure to heavy metals and they have a higher prevalence of some cardiovascular risk factors.

Introduction:

Line 68: hypertension, obesity, diabetes mellitus, tobacco use, dyslipidaemia, ~~obesity,~~ depression, environmental

The word obesity is listed twice, one should be deleted.

Methodology

Line 126: Heavy metals levels ~~was~~  were determined using atomic spectrometer (atomic absorption spectrophotometry?)

Lines 142 -145:

Definition of terms

142……………………………..Elevated total cholesterol was defined as TC >200 mg/dl; low high-density

143 lipoprotein cholesterol (HDL-C) was defined as HDL-C < 40 mg/dl; elevated low-density lipoprotein

144 cholesterol (LDL-C) was defined as LDL-C >130 mg/dl; and elevated triglyceride (TG) was defined as TG 145 >150 mg/dl.2

The units of measurement here are all conventional units; I suggest that the values be also stated for S. I units (in brackets) for the sake of readers who may be used to mainly S.I. units and not conventional units, except if the journal style allows the use of conventional units only.

Ethical consideration:

State the ethical approval number, if required by the journal style.

Results

Lines 170: Majority (state the percentage of this majority) of the study participants were between the age group of 40-60 years, Christians, and married.

Line 173: While at work, about 50% (state the exact percentage) of the auto-mechanics regularly wore overall coat, 156

You can use statetements like: about half of the auto-mechanics (then state the exact percentage in bracket immediately after)

Table 3:

Serum Blood Glucose (mmol/l) 6.30±3.13 5.76±0.96 0.027

This should be simply stated as blood glucose (if whole blood was used as the sample), or plasma glucose, or serum glucose depending on the specific sample used for glucose assay.

Serum NGAL* (ng/ml) 6.03 (2.47) 5.68 (2.10) 0.048

Serum TAC* (in μmol Trolox equiv/L. 392.40 (221.90) 424.50 (288.3) 0.555

For better understanding, authors should state interquartile range (IQR) as a range (Q1 – Q3), and NOT as a whole figure which is the difference between Q3 and Q1.

Discussion:

Lie s196 – 199:

196 The study found that the auto-mechanics had significantly higher

197 prevalence of some of the cardiovascular risk factors as well as elevated serum levels of cadmium

198 compared to the control group who were not auto-mechanics. In addition, a positive association was

199 found between serum levels of some heavy metals, cardiovascular risk factors and renal function.

The facts in these lines have already been overtly stated in the results section; discussion should therefore entail the explicit implications of the findings, and compare with other reports, stating the similarities and differences with possible reason/s for eac of them.

Line 254: About 21% of them smoked cigarette which is similar to 18.3% reported by Ajani

Percentage is a specific figure: the above can be phrased as: About one-fifth (21%), if 21 is the actual percentage, otherwise authors should state the actual percentage in bracket, which may be slightly less than or greater than 21%.

Conclusion:

Lines 288 and 289: There was an association between serum lead and cadmium

levels and some cardiovascular risk factors as well as markers of kidney damage

Conclusion is the take home message (the final inference) of a study, authors should state specifically which cardiovascular risk factors were found to be associated with serum lead and cadmium levels in the study.

.
