# Supplementary material for: Cardiovascular risk factors and kidney function among automobile mechanic and their association with serum heavy metals in Southwest Nigeria: A cross-sectional study
Source: PLoS One. 2023 Oct 11;18(10):e0292364. doi: 10.1371/journal.pone.0292364 (PMC10566715; doi:10.1371/journal.pone.0292364)
Supplement: Attachment — Submitted filename: RESPONSE TO REVIEWERS COMMENTS.docx [file pone.0292364.s005.docx]

| **Reviewer 1** | **Authors’ Response** |
| --- | --- |
| It may not be necessary to repeat the word auto-mechanic (i.e automobile: mechanic). Thus, the  sentence could be simply put as:  This study determined some cardiovascular risk factors, renal function, and their association with  heavy metals among automobile mechanics. | Corrected |
| Method:  Associations between serum lead, cadmium and some cardiovascular risks were determined. | Corrected |
| Results  There were significant positive correlations between serum cadmium level. | Corrected |
| Conclusion:  Auto-mechanics have notable exposure to heavy metals and they have a higher prevalence of  cardiovascular some risk factors.  Auto-mechanics have notable exposure to heavy metals and they have a higher prevalence of  some cardiovascular risk factors. | Corrected |
| Introduction:  Line 68: hypertension, obesity, diabetes mellitus, tobacco use, dyslipidaemia, obesity,. The word obesity is listed twice, one should be deleted. | Corrected |
| Methodology  Line 126: Heavy metals levels was were determined using atomic spectrometer (atomic  absorption spectrophotometry?) | Corrected |
| Lines 142 -145:  Definition of terms  The units of measurement here are all conventional units; I suggest that the values be also stated  for S. I units (in brackets) for the sake of readers who may be used to mainly S.I. units and not  conventional units, except if the journal style allows the use of conventional units only. | Variables expressed in S.I unit (mmol/l) |
| Ethical consideration:  State the ethical approval number, if required by the journal style. | Protocol number provided |
| Lines 170: Majority (state the percentage of this majority) of the study participants were between  the age group of 40-60 years, Christians, and married. | More details provided |
| While at work, about 50% (state the exact percentage) of the auto-mechanics regularly  wore overall coat, 156  You can use statements like: about half of the auto-mechanics (then state the exact percentage  in bracket immediately after) | Revised accordingly |
| Table 3:  Serum Blood Glucose (mmol/l) 6.30±3.13 5.76±0.96 0.027  This should be simply stated as blood glucose (if whole blood was used as the sample), or  plasma glucose, or serum glucose depending on the specific sample used for glucose assay. | Serum deleted as advised |
| Line 173:  Serum NGAL* (ng/ml) 6.03 (2.47) 5.68 (2.10) 0.048  Serum TAC* (in μmol Trolox equiv/L. 392.40 (221.90) 424.50 (288.3) 0.555  For better understanding, authors should state interquartile range (IQR) as a range (Q1 – Q3),  and NOT as a whole figure which is the difference between Q3 and Q1. | IQR provided as Q1-Q3 |
| Line 191-196  The facts in these lines have already been overtly stated in the results section; discussion should  therefore entail the explicit implications of the findings, and compare with other reports, stating  the similarities and differences with possible reason/s for eac of them. | Opening paragraph revised. The results part deleted |
| Line 254: About 21% of them smoked cigarette which is similar to 18.3% reported by Ajani  Percentage is a specific figure: the above can be phrased as: About one-fifth (21%), if 21 is the  actual percentage, otherwise authors should state the actual percentage in bracket, which may be  slightly less than or greater than 21%. | Corrected as suggested |
| Conclusion:  Lines 288 and 289: There was an association between serum lead and cadmium  levels and some cardiovascular risk factors as well as markers of kidney damage  Conclusion is the take home message (the final inference) of a study, authors should state  specifically which cardiovascular risk factors were found to be associated with serum lead and  cadmium levels in the study. | Corrected as suggested |
| **REVIEWER 2** |  |
| Tittle: This phrase “A Cross-sectional Study” should be deleted and supplied under material and  methods.. However, it is helpful to have an idea where the study was carried out as part of the title. | Corrected as suggested |
| Lines 22-24. For the short running title, I suggest “Cardiovascular risk factors and renal function in  auto mechanics in association with serum heavy metals.  . | Corrected as suggested |
| In Abstract Line 39, There was a repetition in the sentence which should be corrected | Corrected as suggested |
| Introduction. Line 88. The authors did not clearly justify the research gap for this study. This should be  highlighted in the introduction. | Justification provided |
| Lines 102 -103. Study population I would like to know how this was achieved i.e. the statement “Any auto-  mechanic with known renal, heart, and liver diseases was excluded&quot;. [ How was this done? Pre or post  sampling?] | Screening was done by taking history and conducting physical examination pre-sampling |
| It would also be interesting to look at the lead and cadmium levels according to years of experience of the  auto mechanics, an interesting result may come out here. | This was not significant. Possibly a large population study follow study will be more revealing |
| How did we exclude the use of supplements like herbal teas that enables detoxifications from being  responsible for the results we are seeing. | We did not exclude this. We have included it as part of limitation of the study |
| Lines131-132. Please state or reference method used for ACR | This has been provided |
| Results Table 3. The repetition of the word serum should be removed having been initially stated in the  methodology section. | Done |
| Discussion. Lines 203-206 This is a rehearse of the results again it is not necessary under discussion.  What is needed here is the implications of the findings. Please comply with this | Done |
| The discussion was lengthy on what was not present in this study among the automobile workers. The oral  sucking of petrol presupposes ingestion from oral mucosa which could lead to elevated cadmium of lead.  This should have been emphasised. | The discussion has been revised by deleting the aspect of what is not present among the automobile workers |
